# Supplementary material for: Extent of illicit cigarette market from single stick sales in Ghana: findings from a cross-sectional survey
Source: BMJ Open. 2023 Mar 21;13(3):e062476. doi: 10.1136/bmjopen-2022-062476 (PMC10040054; doi:10.1136/bmjopen-2022-062476)
Supplement: Author's manuscript [file bmjopen-2022-062476.draft_revisions.pdf]

BMJ Open

BMJ Open is committed to open peer review. As part of this commitment we make the peer review history of every article we publish publicly available.

When an article is published we post the peer reviewers' comments and the authors' responses online. We also post the versions of the paper that were used during peer review. These are the versions that the peer review comments apply to.

The versions of the paper that follow are the versions that were submitted during the peer review process. They are not the versions of record or the final published versions. They should not be cited or distributed as the published version of this manuscript.

BMJ Open is an open access journal and the full, final, typeset and author-corrected version of record of the manuscript is available on our site with no access controls, subscription charges or pay-per-view fees (http://bmjopen.bmj.com).

If you have any questions on BMJ Open's open peer review process please email info.bmjopen@bmj.com

BMJ Open

Estimating the extent of illicit cigarette sales in Ghana: findings from a cross sectional survey

Journal:	*BMJ Open*
Manuscript ID	bmjopen-2022-062476
Article Type:	Original research
Date Submitted by the Author:	03-Mar-2022
Complete List of Authors:	Singh, Arti; Tampere University<br>Ross, Hana; University of Cape Town Research Unit on the Economics of Excisable Products<br>Dobbie, Fiona; The University of Edinburgh College of Medicine and Veterinary Medicine, ;<br>Gallagher, Allen; University of Bath<br>Kinnunen, Tarja; Tampere University<br>Logo, Divine; Ghana Health Service, Research & Development Division-Ghana Health Service; Ministry of Health/Ghana health Service<br>Boateng, Olivia A.; Food and Drug Authority, Tobacco Control and Substance Abuse<br>Gilmore, Anna; University of Bath, Department for Health<br>Bauld, Linda; University of Edinburgh, Usher Institute and UK Centre for Tobacco and Alcohol Studies, College of Medicine and Veterinary Medicine<br>Owusu-Dabo, Ellis; Kwame Nkrumah University of Science and Technology, Department of Global Health
Keywords:	PUBLIC HEALTH, Epidemiology < TROPICAL MEDICINE, STATISTICS & RESEARCH METHODS

SCHOLARONE™
Manuscripts

For peer review only - http://bmjopen.bmj.com/site/about/guidelines.xhtml

I, the Submitting Author has the right to grant and does grant on behalf of all authors of the Work (as defined in the below author licence), an exclusive licence and/or a non-exclusive licence for contributions from authors who are: i) UK Crown employees; ii) where BMJ has agreed a CC-BY licence shall apply, and/or iii) in accordance with the terms applicable for US Federal Government officers or employees acting as part of their official duties; on a worldwide, perpetual, irrevocable, royalty-free basis to BMJ Publishing Group Ltd ("BMJ") its licensees and where the relevant Journal is co-owned by BMJ to the co-owners of the Journal, to publish the Work in this journal and any other BMJ products and to exploit all rights, as set out in our licence.

The Submitting Author accepts and understands that any supply made under these terms is made by BMJ to the Submitting Author unless you are acting as an employee on behalf of your employer or a postgraduate student of an affiliated institution which is paying any applicable article publishing charge ("APC") for Open Access articles. Where the Submitting Author wishes to make the Work available on an Open Access basis (and intends to pay the relevant APC), the terms of reuse of such Open Access shall be governed by a Creative Commons licence – details of these licences and which Creative Commons licence will apply to this Work are set out in our licence referred to above.

Other than as permitted in any relevant BMJ Author's Self Archiving Policies, I confirm this Work has not been accepted for publication elsewhere, is not being considered for publication elsewhere and does not duplicate material already published. I confirm all authors consent to publication of this Work and authorise the granting of this licence.

For peer review only

Estimating the extent of illicit cigarette sales in Ghana: findings from a cross sectional survey

Arti Singh[1, 5], Hana Ross[2], Fiona Dobbie[3], Allen Gallagher[4], Tarja I Kinnunen[5], Divine D. Logo[1], Olivia A. Boateng[6], Anna Gilmore[4], Linda Bauld[3], Ellis Owusu-Dabo[1]

Corresponding author:

Arti Singh
School of Public Health
KNUST, Kumasi
Ghana
Email: artisingh_uk@yahoo.com/arti.singh@tuni.fi

Tel: (233)-244464576

Affiliations

[1]School of Public Health, Kumasi, Ghana

[2]University of Cape Town, South Africa

[3]University of Edinburgh, UK

[4]University of Bath, UK

[5]Tampere University, Finland

[6]Food and Drug Authority, Ghana

Keywords: Illicit, cigarette, Ghana, packs, survey, tobacco

For peer review only

ABSTRACT

Objective This study aims to measure the size of Ghana's illicit cigarette market, to determine the nature and types of illicit cigarettes present in Ghana, and to identify the factors associated with illicit cigarettes sales in Ghana.

Design A Cross-sectional study using empty cigarette packs generated by 1 day's single-cigarette sales collected from cigarette vendors.

Setting Five large cities (Accra, Kumasi, Takoradi, Tamale, and Bolgatanga) and three border towns (Aflao, Paga, and Elubo) in the northern, middle and coastal belt of Ghana

Procedure and Participants Ten areas were randomly selected in each city/town, and all shops selling cigarettes within 1 km of the central point were surveyed.

Outcome measures (1) estimates of the share of illicit packs in the total cigarette sales in Ghana using an empty pack survey method; (2) nature and types of illicit cigarette packs; and (3) factors associated with illicit cigarette sales in Ghana.

Results

Of a total of 4461 packs, 19.5% were found to be illicit. Aflao (Ghana-Togo border) and Tamale (cigarettes coming from Burkina Faso) had the highest percentage of illicit cigarette sales at 98.6% and 45.8% respectively ($p<0.001$). Over half of the illicit packs originated from Togo (51%), followed by Nigeria (14.8%) and then Cote d'Ivoire (10.3%). Adjusted and unadjusted logistic regression models indicated that convenience stores, border towns, and the northern zone had higher odds of illicit cigarette sales.

Conclusion To effectively tackle illicit cigarettes, market surveillance and strengthening supply chain control are required, particularly at the border towns and the northern region of the country.

For peer review only

INTRODUCTION

Illicit tobacco trade continues to remain a threat to global tobacco control efforts. While tobacco consumption is decreasing globally, the African Region is anticipated to experience the world's largest ever increase in a region's number of smokers by 2030 (1) - a projection largely attributed to the rapid population growth, increased advertising by the tobacco industry, and growing tobacco consumption among young people in Africa. Further, the availability and accessibility of cheap, illicit tobacco products is particularly attractive to the region's most vulnerable young population and low-income smokers (2).

Illicit trade of tobacco products is a major public health problem as lower prices of illicit cigarettes lead to increased cigarette consumption (3). Extent of illicit tobacco in the market is difficult to measure, and was estimated to be 11.6% worldwide in 2007 and almost 10% in 2015 (3), and these figures are higher for low and middle income countries (LMICs) including those in the African Region. In response to the threat posed by illicit tobacco trade, the WHO FCTC Protocol to Eliminate Illicit Trade in Tobacco Products (hereby referred to as "the Protocol") entered into force in 2018 (4). This Protocol gives countries an opportunity to prevent tobacco-related morbidity and mortality by enhancing tobacco supply chain control. Countries that ratify the Protocol commit themselves to adopting a variety of measures, including track and trace systems to prevent and counter illicit trade.

Ghana, one of the first countries to ratify the World Health Organization's (WHO) Framework Convention on Tobacco Control (FCTC) in 2004, has made some significant progress in tobacco control such as introducing an early advertising ban (1982), the passage of the Tobacco Control Act (in 2012), banning of singlestick sales (2017), introduction of mandatory graphic health warnings (2018) and tax stamps on

For peer review only

tobacco products (2018) and more recently the ratification of the Protocol in October 2021 (5). Despite this progress, cigarettes continue to remain cheap and affordable in Ghana (1). The total excise tax on tobacco products in Ghana accounts for only 31.8% of the average retail price, far below the 70% benchmark set by the World Health Organization (WHO) (1). Although, Ghana does not have an active tobacco industry (British American Tobacco (BAT) ceased its local production in 2006), BAT continues to dominate sales of cigarettes and remains the dominant importer of cigarettes into the country via its manufacturing sites in Ibadan and Zaria in Nigeria (5). The distribution networks of Ghana's leading tobacco companies are well organised in Ghana's major urban cities including Greater Accra, Takoradi, Kumasi, and Tamale. The point of sale of tobacco products including cigarettes in Ghana is mainly via the traditional grocery retailers (also known as convenience or provision stores), street vendors, kiosks and drinking bars most of which may be unregulated and without a license to operate (6).

An important challenge that exists in many African countries, including Ghana, is that most governments do not measure the size of illicit tobacco market nor analyze its features on a regular basis. To fully benefit from the Protocol, policymakers and implementers seek to connect its normative guidance with empirical data and analysis on countries' illicit tobacco trade. This means that they are not able to monitor and adapt measures to control illicit trade (9). In light of the tobacco industry's use of illicit trade to oppose tobacco control such as including tax increases (7), it is important to understand the scope and nature of the illicit tobacco trade. To date, there have no scientific studies to estimate the size of the illicit cigarette market in Ghana (1). The only available estimates are those produced by the Euromonitor that reports an illicit cigarette market accounting for 39% of total cigarette sales in 2018 (up from 35% in 2017) (10). Estimates by Euromonitor have been criticized for being unreliable and

For peer review only

inconsistent, and for lacking independence due to Euromonitor entering into business contracts with Philip Morris International (PMI) (5,11). The objectives of this study were to measure the size of Ghana's illicit cigarette market using an empty pack survey method to determine the nature and types of illicit cigarettes present in Ghana, and to identify the factors associated with illicit cigarettes sales in Ghana.

METHODS

Study sites

A cross sectional study was conducted during the months of August 2020 to January 2021 in four major cities in Ghana (Accra, Tamale, Kumasi, Takoradi and Bolgatanga) and four border towns (Aflao, Paga and Elubu) across the three zones of Ghana (Northern, Middle and Coastal) (Figure 1). These districts were selected to represent socioeconomic, cultural and geographical diversity.

Insert figure 1

Research design

A modified approach based on the analysis of empty cigarette packs collected directly from retailors was used. This method was adapted from similar studies in India (8) and Bangladesh (9) and is particularly useful in countries where single stick sales are a common practice. Within each large city or border town, up to 10 smaller geographical areas were selected using Ghana Post Codes. A central point (such as a government building, market place or taxi station) was determined in each of them for retailor pack collection. A team of four research assistants and a coordinator walked 1 km along both sides of a busy street (0.5 km forward and 0.5 km back) starting from the central point to identify tobacco retailors. All retailers identified were provided with verbal and written information about the study and requested to sign a consent form if they agreed to participate. Following consent being obtained, an empty bag with a unique identifier was given to retailers and they were asked to deposit all cigarette packs emptied

For peer review only

throughout the day as a result of single sticks of cigarette sales in the bag provided. The bags were collected back from the retailors at the end of a 24-hour period and retailors were given a small monetary reward (up to USD 10) commensurate with the number of packs provided. Consenting retailors also participated in a 20-25 minutes survey on illicit cigarette sales, common brands, and pricing of cigarettes sold each day. The sample size equation to obtain the minimum number of packs collected from each selected city/town was adapted from a toolkit for measuring illicit tobacco in LMICs (10). We obtained a minimum sample size of 2600 packs to estimate a size of 25% illicit cigarette sales with a 95% level of confidence.

Classification of packs

Empty cigarette packs were cleaned and assigned unique IDs, and were analysed and their characteristics recorded. Pack data included the brand name, country of origin, the presence of graphical and/or textual health warnings, the language of the warning, the pack size (10/20 stick pack), and compliance of these warning messages with existing packaging requirement for Ghana. A conservative definition to classify an illicit cigarette pack (packs on which appropriate duties have not been paid) in Ghana according the Food and Drugs Authority (11), includes at least one of the following attributes:

(a) Absence of authentic tax stamps;

(b) Absence of textual and pictorial warnings;

(c) Absence of the inscription "*FOR SALE IN GHANA ONLY*" displayed on the side panel of the product pack and

(d) Health warnings not in English

Trained research assistants evaluated tax stamp authenticity using the tax stamp mobile application developed by the Ghana Revenue Service (12).

For peer review only

Analysis

Data were first entered into excel, cleaned and analyzed via R studio version 1.4.1717. The unit of analysis was each cigarette pack. Continuous variables such as price/pack were changed to categorical (low and high price category) for 2-7 GHC and 8-14 GHC respectively for purposes of analysis (1USD=6GHC). Descriptive information was reported as frequencies and percentages for city, country zone (northern, middle and coastal zones), retail shop type (drinking bars, convenience stores and kiosks), border and non-border towns, country of origin and illicit and licit cigarette. Pack characteristics such as pictorial health warning (absent/present), textual health warning (absent/present), warning labels in English (absent/present), tax stamps (absent/present) and "*for sale in Ghana*" sign (absent/present) were captured. The relationship between illicit tobacco and the categorical variables (city type, country zone, type of shops, border and non-border town, price/packs, cigarette brand and country of origin) were first studied using $\chi 2$ or Fisher's exact test (when the number in the table was <6). Due to the binary nature of the outcome variable (licit/illicit), simple and multiple logistic regression was performed to evaluate the unadjusted and adjusted predictive values of the potential confounding variables respectively based on existing literature (13,14). The results are presented as odds ratios (OR) with a 95% confidence interval, with significance set at an alpha level of 5% ($p \leq 0.05$).

Patient and public Involvement

No patient involved

RESULTS

A total of 425 retailors were approached for the study, of whom 384 (90%) consented to collect packs and participate in the survey. An average of 12 cigarette packs were collected by in a 24-hour period. A total of 4461 packs were collected from 384 retailors in the selected cities and towns. All retailors (100%) in the study sold single

For peer review only

sticks (100%). A total of 871 out of 4461 (19.5%) packs were classified as illicit based on the criteria for classification approved by the FDA. Over half of the packs (58.6%) were collected from drinking bars of which 18.2% were illicit (Table 1). A third (30.6%) of the packs collected from the northern zone of Ghana were illicit and almost seven out of 10 (68.5%) packs from the border towns were illicit. Almost all the packs collected from Aflao (Ghana-Togo border) were illicit (98.6%), followed by Tamale (45.8%) and the Paga/Hamele (Ghana-Burkina Faso border) (26.6%) and Elubu (21.1%) (Ghana - Cote d'Ivoire border). In terms of the retail selling points, three out of 10 (29%) packs collected from convenience stores were illicit, followed by drinking bars (18.2%) ($p<0.001$). Over 60% of the packs collected within the price category of 2-7 GHC were illicit. The most common brand of cigarettes sold in Ghana is Rothmans Kingsize, London Brown/White and Pallmall (Figure 2).

Insert Figure 2

Of all the 871 illicit packs, the most common brands were Business Royal (24.1%), followed by Fine (20.8%) and Oris (12.3%). All packs from 555 and London Brown/White (manufactured by BAT) were licit (100%) (Table 1).

For peer review only

Table 1: **Determinants of illicit cigarette sale in Ghana**

	**Illicit cigarette packs (n=871)**	**Licit cigarettes packs (n=3590)**	**Total**
Country Zone			
Northern	368 (30.6)	835 (69.4)	1203 (100)
Middle	8 (1.2)	656 (98.8)	664 (100)
Coastal (south)	495 (19.1)	2099 (80.9)	2594 (100)
*P-value**		*0.000*	
Border/non-border			
Border	493 (68.5)	227 (31.5)	720 (100)
Non-border	378 (10.1)	3363 (89.1)	3741 (100)
*P-value**		*0.000*	
City/town (border/non border)			
Accra (non border)	17 (1.5)	1147 (98.5)	1164 (100)
Kumasi (non border)	8 (1.2)	651 (98.8)	659 (100)
Takoradi (non border)	1 (0.1)	767 (99.9)	768 (100)
Bolgatanga (non border)	7 (1.8)	390 (98.2)	397 (100)
Tamale (non border)	345 (45.8)	408 (54.2)	753 (100)
Elubu (Cote d'ivoire border)	44 (21.1)	165 (78.9)	209 (100)
Paga/Hamele (Burkina Faso border)	16 (26.6)	42 (72.4)	58 (100)
Aflao (Togo border)	433 (98.6)	20 (1.4)	453 (100)
P-value*		0.000	
Shop type			
Drinking bar	477 (18.2)	2139 (81.8)	2616 (100)
Kiosks	31 (5.2)	563 (94.8)	594 (100)
Convenience stores	363 (29.0)	888 (71.0)	1251 (100)
*P-value**		*0.000*	
Price/pack (GHC)			
Low price (2-7)	778 (61.2)	494 (38.8)	1272 (100)
High price (8-14)	93 (2.9)	3096 (97.1)	3189 (100)
P-value*		0.000	
Cigarette brand (manufacturer)			
555 (BAT)	0 (0)	190 (100)	190(100)
London Brown/White (BAT)	0 (0)	928 (100)	928 (100)
Pallmall (BAT)	70 (14.2)	433 (85.8)	494 (100)
Business Royal (Independent Tobacco Inc)	210 (70.0)	90 (30.0)	300 (100)
Fine (unknown)	181 (78.3)	50 (21.6)	231 (100)
Rothmans Kingsize (BAT)	29 (1.6)	1798 (98.4)	1827 (100)
Oris (Oriental General Trading Inc)	107 (81.1)	35 (18.9)	132 (100)
Rothmans Royals (BAT)	99 (86.1)	20 (13.9)	115 (100)
Gold Seal (China Tobacco)	85 (91.4)	8 (8.6)	93 (100)
Tusker (BAT)	29 (100)	0 (0)	29 (100)
Others (Fisher, menthol, Cherry etc.)	61(50.0)	61 (50.0)	122 (100)
*P-value**		*0.000*	

*P-value based on $\chi 2$ or Fisher's exact test

For peer review only

Majority of the illicit packs were characterized by absence of tax stamps (94.3%), '*for sale in Ghana'* sign (92.2%) and warning labels in English (77.3%).

Almost all the packs collected were the 20-stick pack (98.2%). The average price/pack of the 20-stick packs was 8.5 GHC and that for 10-stick was 3.3 GHC. Illicit packs had an average price/pack of 5.4 GHC (SD 1.5, range 2-12 GHC) whilst licit pack was 9.1 GHC (SD 2.1, range 2-14 GHC). Close to half of the illicit packs originated from Togo (51%), followed by Nigeria (14.8%) and then Cote d'Ivoire (10.3 %). About 1.5% of packs that were destined for Ghana were classified as illicit as the packs did not conform to the current labeling requirements as approved by FDA.

Table 2 shows the results from adjusted and unadjusted logistic regression of the factors associated with illicit cigarette sales in Ghana. The odds of illicit cigarette sales were 1.8 folds and 2.68 folds higher in convenience stores as compared to drinking bars in the unadjusted and adjusted models respectively (Table 2). Also, the sale of illicit cigarettes was 19.32 and 69.69 odds higher in border towns as compared to non-border towns in both the adjusted and unadjusted models respectively. The middle and coastal country zones had lower odds of illicit cigarettes sales than the northern zones in both the unadjusted and adjusted regression models respectively.

Table 2: Unadjusted and unadjusted factors for illicit cigarette sales in Ghana

Variable	Unadjusted		Adjusted	
	OR	95% CI	OR	95% CI
Retail shop type				
Drinking bars	1		1	
Kiosks	0.25	0.17 - 0.35	0.26	0.15-0.45
Convenience stores	1.83	1.57-2.15	2.68	1.78-4.05

Country Zone				
Northern	1		1	
Middle	0.03	0.01-0.05	0.06	0.03 -0.12
Coastal	0.54	0.46-0.63	0.17	0.11-0.25
Border/non border towns				
Non-border town	1		1	
Border town	19.32	16.0-23.4	69.69	51.45-96.05

DISCUSSION

This study found out that 19.5% of the packs collected were illicit of the total 4461 packs. Majority of the illicit packs were reported from Aflao (Ghana-Togo border) (98.6%) and Tamale (northern zone with cigarettes coming from Burkina Faso) (45.8%). Close to half of the illicit packs originated from Togo (51%), followed by Nigeria (14.8%) and then Cote d'Ivoire (10.3 %). The most common brand of cigarettes sold in Ghana was from BAT including Rothmans Kingsize, London Brown/White and Pallmall. One out four of the illicit packs belonged to Business Royal (Independent Tobacco Company), a fifth were from Fine (unknown company) and about one out of ten were from Oris brand (Oriental Genral Trading). Absence of tax stamps, '*for sale in Ghana'* sign and warning labels in English were among the most common characteristic of the illicit packs. Adjusted and unadjusted logistic regression models indicated that convenience stores, border towns and northern zone of the country had higher odds of sale of illicit cigarettes in Ghana.

Our study provides an objective measure and describes the nature of the illicit cigarette market. This plays a critical role in developing comprehensive and effective tobacco control policies, particularly in countries within SSA such as Ghana, where

For peer review only

data on illicit cigarettes sales is lacking. The direct interaction with retailers also allowed us to obtain additional information about the price, the daily retail volume and pack characteristics of the cheapest cigarette brand sold in the store by each vendor. Our illicit cigarette estimate (19.5%), is lower than the estimates of the Euromonitor (37% in 2018) (15). This is not surprising based on the lack of transparency in the Euromonitor data and their funding from the tobacco industry (TI) (15). The TI is known for quoting high estimates of the illicit market as a means of deterring governments from imposing tobacco tax increases, which contributes to ineffective tobacco control and lost opportunities for the governments to collect more revenue.

There are various methods to assess the extent of illicit tobacco in any country, such as measuring the difference between consumption and tax paid sales (gap analysis), interviewing smokers, examination of littered cigarette packs and econometric modeling (16). We employed the empty pack methodology, which is particularly suitable in countries with single stick sales, such as in India (8), Pakistan (17), Bangladesh (9) and Argentina (18). Indeed, in our study, despite a ban on single stick sales, all retailors (100%) sold single sticks, calling for enforcement of the ban. Our estimates of illicit cigarette sales are similar to countries with a higher tobacco use prevalence such as Pakistan (17.8%) and Argentina (13.7%) that used a similar methodology (17,18). Despite the lack of estimates of illicit cigarettes from many countries in the African Region, countries such as South Africa, Kenya, Gambia and Nigeria have available estimates of their illicit market. Our estimates were found to be lower than South Africa (over 30% of the total market in 2017) (19), Nigeria (26.3%) (20) and Kenya (26%) (21) but higher than the Gambia (8.6%) (22). With the ratification of the Protocol in Ghana, and estimates suggesting 1 out 5 cigarette packs

For peer review only

to be illicit, there is an urgent need for governments to address this by fully implementing the recently ratified protocol (which has specific requirements to improve traceability of tobacco products and increase tobacco industry accountability).

British American Tobacco (BAT) continues to dominate sales of cigarettes as evidenced by the most common cigarettes sold in Ghana (Rothmans Kingsize, London Brown/White and PallMall). This is largely due to the company's long history in Ghana. While the company ceased domestic production in 2006, it remains the dominant importer of cigarettes into the country. There are also very low-priced brands available, such as BAT's Tusker brand (of which all packs were illicit). While, all packs from London Brown/White were found to be licit, about 14% of PallMall and 1.6% of Rothmans Kingsize were illicit, demonstrating the industry's involvement in illicit trade. Further, the small-scale convenience stores were found to be a major selling point of illicit cigarettes. These are legally operating, widely available settings to the low-income Ghanaian smoker (who prefers to buy single stick) widely available in both rural and urban locations. Convenience stores were also found to have higher odds of illicit cigarette sales as compared to drinking bars in both the adjusted and unadjusted logistic regression models, indicating that it is a significant predictor of illicit cigarette sales in the country.

Geography was found to play an important role in the illicit cigarette market in Ghana. A third of the packs collected from the northern zone of the country were found to be illicit. According to the Euromonitor, the north of Ghana sees particularly strong illicit trade, with most smuggling from Burkina Faso finding their way to this region (15). This could also be strongly linked to the high smoking prevalence in the region as compared to other regions (23). Similarly, border towns were also found to

For peer review only

be strong predictors of illicit cigarette sales. Six out of 10 packs collected from border towns were illicit and almost 100% of the packs collected from Aflao (Ghana-Togo border), and close to half of the packs from Tamale (large city in Northern Ghana linked to Burkina Faso) were found to be illicit. This finding is consistent with other studies in Vietnam (24) and Georgia (25) where border towns were more vulnerable to illicit trade. This finding reinforces the need for strengthening patrolling and border control in addition to building capacity and training for authorities belonging to customs, police and immigration. The illicit cigarettes originated from Togo (51%), followed by Nigeria (14.8%) and then Cote d'Ivoire (10.3 %). Nigerian products are mostly smuggled in via Togo and most products smuggled in from Togo originate from BAT's Nigerian operations, with lower taxes in Nigeria enabling these to be sold at a lower price in Ghana. As observed in our study, the health warnings on cigarette packages are also in French, indicating a French-speaking West African source country.

In terms of pricing of cigarettes, illicit packs were found to be almost 50% cheaper than licit packs. Africa in general, lags behind other regions (such as European and the Americas) in implementing strong tobacco tax policies (1). Close to 90% of the illicit packs were found in the low price category (2-7 GHC). In Ghana, the total excise tax on tobacco products accounts for only 31.8% of the average retail price, far below the 70% benchmark set by the WHO with no significant change in the affordability of cigarettes since 2010 (1). Over half of the smuggled cigarettes in the study originated from Togo (where cigarette are less affordable as compared to Ghana). Around Ghana's neighboring countries, the total excise tax on tobacco products account for 41.4%, 35.1% and 34.5% of the average retail price in Togo, Nigeria and Cote d'Ivoire respectively. Although, the TI argues that smuggling is

For peer review only

heavily influenced by cross-border price differences, and higher taxation increases its profitability, this is highly debatable (26). Available data shows that price levels do not predict levels of illicit trade and the relationship between taxation and smuggling is more complex than it appears (27). An important point to consider is that, regional cooperation and coordination of tobacco tax and price levels remains a powerful strategy to consider in order to weaken the link between tobacco tax increases and illicit trade. Limiting tax discrepancies between neighboring countries can reduce arbitrage opportunities for smugglers at borders. Thus, it is important to intensify implementation efforts for such coordinated measures, for example within the Economic Community of West African States (ECOWAS) region to harmonize tax options (28) .

Our study findings should be considered in the light of some limitations. First, despite the wide geographical dispersion in the three zones of the country (northern, middle and coastal), the representativeness to the country is limited. Also, as data was collected during COVID-19 lockdown period in Ghana and we could not explore other border towns that were planned due to pertaining restrictions. Secondly, the empty pack collection relies on retailers to provide us with all the empty packs sold that day. It could be possible that retailers would want to hide the illegal packs, which could underestimate our findings. Nevertheless, retailors were motivated with a monetary reward commensurate with the number of packs collected, which, to an extent, mitigated this issue. Third, our survey was able to collect empty cigarette packs from retailers mainly from drinking bars, kiosks and convenience stores. Thus street hawkers and dealers, if any, who are on the move and sell cigarettes are not covered by the survey.

CONCLUSION

For peer review only

361 Our study found a total of 19.5% illicit packs in the entire sample of packs collected
362 across the eight cities in Ghana. Our estimate of the illicit cigarette market share is
363 below with the estimates provided by the Euromonitor. This study provides valuable
364 information for policymakers and law enforcement in the region and bringing to light
365 the inadequacy of the current monitoring and regulatory activities of the FDA and
366 customs. Our findings have three important policy implications; first, the regulatory
367 body and the focal point for tobacco control in Ghana (FDA) in collaboration with the
368 customs, police and immigration, should strengthen the supply chain control and
369 market surveillance at retail points in the towns and cities, particularly those close to
370 the border in the northern and coastal zones of the country, aside from border
371 monitoring and transportation tracing. Secondly, among the ECOWAS member
372 states, there is a need to harmonise excise and taxation levels on tobacco across West
373 Africa. This could reduce the problem of smuggled goods, as the competitive price
374 advantage for some ECOWAS member states would be removed. Finally, with the
375 introduction of Tax Stamp Policy since March 2018, Ghana should also consider the
376 implementation of a supply chain control that resembles a track and trace system (like
377 Kenya), independent of any industry influence to effectively monitor the illicit
378 market.

379

What this paper adds

- There is an absence of an independent and scientifically verifiable estimate of illicit cigarette sales in Ghana.
- The study provides estimates of the share of illicit packs in the total cigarettes sales in Ghana.
- One out of five cigarette packs sold in Ghana is illicit, using a rigorous empty pack survey methodology in eight cities/towns including border towns.

For peer review only

- We highlight the urgent action needed in Ghana to strengthen supply chain control and border control to effectively combat illicit trade.

ACKNOWLEDGEMENTS

We would like to thank all the retailors that provided information and packs for this survey.

CONTRIBUTORS

AS drafted the initial version of the manuscript. HR, FD, TK and AG contributed to the revision of the manuscript for important intellectual content and final approval. All other authors reviewed the final draft for approval.

COMPETING INTEREST

None declared

FUNDING

This work was supported by the Medical Research Council [grant number MR/P027946/2] with funding from the Global Challenges Research Fund and with additional funding from the University of Edinburgh's Scottish Funding Council Global Challenges Research Fund (GCRF) allocation.

Ethics Approval

The study protocol was approved by the Committee on Human Research, Publication and Ethics (Reference number: CHRPE/AP/441/18) and the University of Bath's Research Ethics Approval Committee for Health (REACH) (EP 19/20 063).

Data sharing statement

For peer review only

The data are owned and shared by the Tobacco Control Capacity Program (TCCP) and the School of Public Health, KNUST, Ghana. Requests for data sharing can be made to artisingh_uk@yahoo.com.

REFERENCES

1. WHO. Who Report on the Global Tobacco Epidemic, 2021. 2021;
2. Confronting illicit tobacco trade: a global review of country experiences.
3. Joossens L, Merriman D, Ross H et al IU against T and L. How eliminating the global illicit cigarette trade would increase tax revenue and save lives. 2009 [cited 2022 Feb 3];30. Available from: https://theunion.org/technical-publications/how-eliminating-the-global-illicit-cigarette-trade-would-increase-tax-revenue-and-save-lives
4. Transnational Alliance to Combat Illicit Trade. Mapping the Impact of Illicit Trade on the Sustainable Development Goals. 2019; Available from: www.tracit.org/publications.html
5. Singh A, Owusu-Dabo E, Mdege N, McNeill A, Britton J, Bauld L. A situational analysis of tobacco control in Ghana: progress, opportunities and challenges. Journal of Global Health Reports. 2020;

6. Tobacco in Ghana | Market Research Report | Euromonitor [Internet]. [cited 2021 Aug 17]. Available from: https://www.euromonitor.com/tobacco-in-ghana/report

7. Joossens L, Merriman D, Ross H, Raw M. The impact of eliminating the global illicit cigarette trade on health and revenue. Addiction. 2010 Sep 1;105(9):1640–9.

8. John RM, Ross H. Illicit cigarette sales in Indian cities: findings from a retail survey. Tob Control. 2018 Nov 1;27(6):684–8.

9. Abdullah SM, Huque R, Bauld L, Ross H, Gilmore A, John RM, et al. Estimating the Magnitude of Illicit Cigarette Trade in Bangladesh: Protocol for a Mixed-Methods Study. International Journal of Environmental Research and Public Health 2020, Vol 17, Page 4791 [Internet]. 2020 Jul 3 [cited 2022 Jan 5];17(13):4791. Available from: https://www.mdpi.com/1660-4601/17/13/4791/htm

10. Michal S, Guillermo P, Blecher E. A Toolkit on Measuring Illicit Trade in Tobacco Products. 2020;

11. Welcome to Ghana Food And Drugs Authority | Home [Internet]. [cited 2022 Jan 5]. Available from: http://www.fdaghana.gov.gh/index.php

12. GRA introduces Ghana Tax Stamp Authenticator mobile app [Internet]. [cited 2022 Feb 23]. Available from: https://www.ghanaweb.com/GhanaHomePage/business/GRA-introduces-Ghana-Tax-Stamp-Authenticator-mobile-app-771674

13. Vellios N, Van Walbeek C, Ross H. Measuring the illicit cigarette market in the absence of pack security features: A case study of South Africa. Tobacco Control. 2021 Feb 25;

For peer review only

14. John RM, Ross H. Consumption of illicit cigarettes in India: findings from a retail survey. Tobacco Induced Diseases. 2018;

15. Profile I. Tobacco in. 2011;(October):1–32.

16. A Toolkit on Measuring Illicit Trade in Tobacco Products | Tobacconomics [Internet]. [cited 2022 Jan 5]. Available from: https://tobacconomics.org/research/a-toolkit-on-measuring-illicit-trade-in-tobacco-products/

17. Khan A, Dobbie F, Siddiqi K, Ansaari S, Abdullah SM, Iqbal R, et al. Illicit cigarette trade in the cities of Pakistan: comparing findings between the consumer and waste recycle store surveys. Tobacco Control [Internet]. 2021 Apr 15 [cited 2021 Dec 30];1–7. Available from: https://tobaccocontrol.bmj.com/content/early/2021/04/14/tobaccocontrol-2020-056386

18. Pizarro ME, Giacobone G, Shammah C, Stoklosa M. Illicit tobacco trade: empty pack survey in eight Argentinean cities. Tobacco Control [Internet]. 2021 Apr 8 [cited 2021 Dec 30];0:1–7. Available from: https://tobaccocontrol.bmj.com/content/early/2021/04/07/tobaccocontrol-2020-056405

19. Vellios N, van Walbeek C, Ross H. Illicit cigarette trade in South Africa: 2002–2017. Tobacco Control [Internet]. 2020 Oct 1 [cited 2021 Dec 30];29(Suppl 4):s234–42. Available from: https://tobaccocontrol.bmj.com/content/29/Suppl_4/s234

20. Adeniran AP, Ekeruche MA, Castradori M. Illicit Tobacco in Nigeria [Internet]. Centre for the Study of the Economies of Africa (CSEA); 2020 [cited 2021 Dec 23]. Available from:

For peer review only

https://www.africaportal.org/publications/illicit-tobacco-nigeria/
21. Ross H. Tracking and tracing tobacco products in Kenya. Preventive Medicine. 2017 Dec 1;105:S15–8.
22. Chisha Z, Janneh ML, Ross H. Consumption of legal and illegal cigarettes in the Gambia. Tob Control [Internet]. 2020;29:254–9. Available from: http://tobaccocontrol.bmj.com/
23. Yawson AE, Baddoo A, Hagan-Seneadza NA, Calys-Tagoe B, Hewlett S, Dako-Gyeke P, et al. Tobacco use in older adults in Ghana: Sociodemographic characteristics, health risks and subjective wellbeing. BMC Public Health. 2013;
24. Nguyen HTT, Giang LT, Pham TN. Empirical analysis on the illicit trade of cigarettes in Vietnam. Tobacco Control [Internet]. 2020 Oct 1 [cited 2021 Dec 30];29(Suppl 4):s281–6. Available from: https://tobaccocontrol.bmj.com/content/29/Suppl_4/s281
25. Little M, Ross H, Bakhturidze G, Kachkachishvili I. Illicit tobacco trade in Georgia: prevalence and perceptions. Tobacco Control [Internet]. 2020 Oct 1 [cited 2021 Dec 30];29(Suppl 4):s227–33. Available from: https://tobaccocontrol.bmj.com/content/29/Suppl_4/s227
26. Gallagher AWA, Evans-Reeves KA, Hatchard JL, Gilmore AB. Tobacco industry data on illicit tobacco trade: a systematic review of existing assessments. Tob Control. 2019 May 1;28(3):334–45.
27. van Walbeek C. Measuring changes in the illicit cigarette market using government revenue data: the example of South Africa. Tob Control. 2014;23(E1).
28. Campaign for Tobacco-free Kids. Highest Excise and Total Tax Burden.

For peer review only

CIGARETTE TAXES IN AFRICAN COUNTRIES. 2020.

Legends for figures

Figure 1: Location of the eight cities for pack collection in Ghana

Figure 2: Cigarette brands sold in Ghana

For peer review only

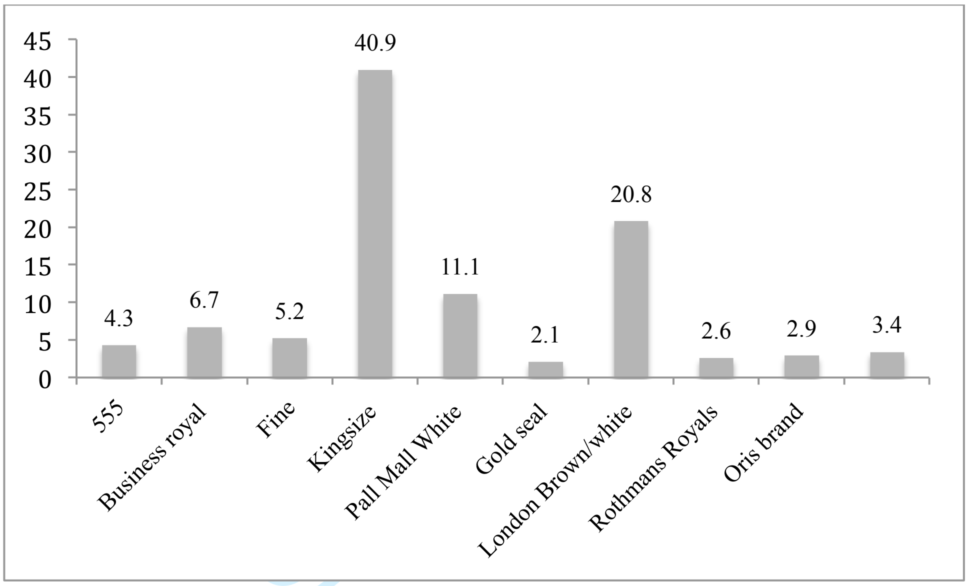

For peer review only

BMJ Open

Extent of illicit cigarette market from single stick Sales in Ghana: findings from a cross-sectional survey

Journal:	*BMJ Open*
Manuscript ID	bmjopen-2022-062476.R1
Article Type:	Original research
Date Submitted by the Author:	10-Aug-2022
Complete List of Authors:	Singh, Arti; Tampere University<br>Ross, Hana; University of Cape Town Research Unit on the Economics of Excisable Products<br>Dobbie, Fiona; The University of Edinburgh College of Medicine and Veterinary Medicine, ;<br>Gallagher, Allen; University of Bath<br>Kinnunen, Tarja; Tampere University<br>Logo, Divine; Ghana Health Service, Research & Development Division-Ghana Health Service; Ministry of Health/Ghana health Service<br>Boateng, Olivia A.; Food and Drug Authority, Tobacco Control and Substance Abuse<br>Gilmore, Anna; University of Bath, Department for Health<br>Bauld, Linda; University of Edinburgh, Usher Institute and UK Centre for Tobacco and Alcohol Studies, College of Medicine and Veterinary Medicine<br>Owusu-Dabo, Ellis; Kwame Nkrumah University of Science and Technology, Department of Global Health
**Primary Subject Heading**:	Public health
Secondary Subject Heading:	Epidemiology, Global health, Public health, Smoking and tobacco
Keywords:	PUBLIC HEALTH, Epidemiology < TROPICAL MEDICINE, STATISTICS & RESEARCH METHODS

SCHOLARONE™
Manuscripts

I, the Submitting Author has the right to grant and does grant on behalf of all authors of the Work (as defined in the below author licence), an exclusive licence and/or a non-exclusive licence for contributions from authors who are: i) UK Crown employees; ii) where BMJ has agreed a CC-BY licence shall apply, and/or iii) in accordance with the terms applicable for US Federal Government officers or employees acting as part of their official duties; on a worldwide, perpetual, irrevocable, royalty-free basis to BMJ Publishing Group Ltd ("BMJ") its licensees and where the relevant Journal is co-owned by BMJ to the co-owners of the Journal, to publish the Work in this journal and any other BMJ products and to exploit all rights, as set out in our licence.

The Submitting Author accepts and understands that any supply made under these terms is made by BMJ to the Submitting Author unless you are acting as an employee on behalf of your employer or a postgraduate student of an affiliated institution which is paying any applicable article publishing charge ("APC") for Open Access articles. Where the Submitting Author wishes to make the Work available on an Open Access basis (and intends to pay the relevant APC), the terms of reuse of such Open Access shall be governed by a Creative Commons licence – details of these licences and which Creative Commons licence will apply to this Work are set out in our licence referred to above.

Other than as permitted in any relevant BMJ Author's Self Archiving Policies, I confirm this Work has not been accepted for publication elsewhere, is not being considered for publication elsewhere and does not duplicate material already published. I confirm all authors consent to publication of this Work and authorise the granting of this licence.

For peer review only

Extent of illicit cigarette market from Single Stick Sales in Ghana: findings from a cross sectional survey

Arti Singh[1, 5], Hana Ross[2], Fiona Dobbie[3], Allen Gallagher[4], Tarja I Kinnunen[5], Divine D. Logo[1], Olivia A. Boateng[6], Anna Gilmore[4], Linda Bauld[3], Ellis Owusu-Dabo[1]

Corresponding author:

Arti Singh
School of Public Health
KNUST, Kumasi
Ghana
Email: artisingh_uk@yahoo.com/arti.singh@tuni.fi

Tel: (233)-244464576

Affiliations

[1]School of Public Health, Kumasi, Ghana

[2]University of Cape Town, South Africa

[3]University of Edinburgh, UK

[4]University of Bath, UK

[5]Tampere University, Finland

[6]Food and Drug Authority, Ghana

Keywords: Illicit, cigarette, Ghana, packs, survey, tobacco

For peer review only

ABSTRACT

Objective This study aims to measure the extent of illicit cigarette consumption from single stick sales, to determine the nature and types of illicit cigarettes present in Ghana, and to identify the factors associated with illicit cigarettes consumption in Ghana.

Design A Cross-sectional study using empty cigarette packs generated by 1 day's single stick cigarette sales collected from cigarette vendors.

Setting Five large cities (Accra, Kumasi, Takoradi, Tamale, and Bolgatanga) and three border towns (Aflao, Paga/Hamele and Elubo) in the northern, middle and coastal belt of Ghana.

Procedure and Participants Ten areas were randomly selected in each city/town, and all shops selling cigarettes within 1 km of the central point were surveyed.

Outcome measures (1) estimates of the share of illicit cigarette packs in the total cigarette sales from vendors selling single stick cigarettes in Ghana; (2) nature and types of illicit cigarette packs; and (3) factors associated with illicit cigarette sales in Ghana.

Results

Of a total of 4461 packs, about 20% (95% CI: 18.34-20.66) were found to be illicit. Aflao (Ghana-Togo border) and Tamale (Ghana-Burkina Faso border) had the highest percentage of illicit cigarette sales at 99% and 46% respectively ($p<0.001$). Over half of the illicit packs originated from Togo (51%), followed by Nigeria (15%) and then Cote d'Ivoire (10%). Adjusted and unadjusted logistic regression models indicated that convenience stores, border towns, pack price and the northern zone had higher odds of illicit cigarette sales.

For peer review only

Conclusion To effectively tackle illicit cigarettes, market surveillance and strengthening supply chain control are required, particularly at the border towns and the northern region of the country.

Strengths and limitations of this study

- This study provides the first independent estimate of the share of illicit cigarette consumption in five big cities and four border towns in Ghana using a new method suitable for countries with prevalent single-cigarette sales.
- The empty pack survey required little time and resources to conduct.
- Some retailers may not provide all the packs over the last 24 hours, which could underestimate our findings.
- The study was limited to five cities and three border towns and is not representative of illicit cigarettes sales in Ghana as a whole.

For peer review only

INTRODUCTION

Illicit tobacco trade continues to remain a threat to global tobacco control efforts. While tobacco consumption is decreasing globally, rapid population growth, increased advertising by the tobacco industry, and growing tobacco consumption among young people in Africa may result in increased number of smokers in the region (1). Further, the availability and accessibility of cheap, illicit tobacco products is particularly attractive to the region's most vulnerable young population and low-income smokers (2).

Illicit trade of tobacco products is a major public health problem as lower prices of illicit cigarettes lead to increased cigarette consumption (3). Despite the difficulties in measuring the extent of illicit tobacco in the market, available estimates indicates that it was about 11.6% worldwide in 2007 and almost 10% in 2015 (3), and these figures are higher for low and middle income countries (LMICs) including those in the African Region. In response to the threat posed by illicit tobacco trade, the WHO FCTC

Protocol to Eliminate Illicit Trade in Tobacco Products (hereby referred to as “the Protocol”) entered into force in 2018 (4). This Protocol gives countries an opportunity to prevent tobacco-related morbidity and mortality by enhancing tobacco supply chain control. Countries that ratify the Protocol commit themselves to adopting a variety of measures, including track and trace systems to prevent and counter illicit trade.

Ghana, one of the first countries to ratify the World Health Organization’s (WHO) Framework Convention on Tobacco Control (FCTC) in 2004, has made some significant progress in tobacco control such as introducing an early advertising ban (1982), the passage of the Tobacco Control Act (in 2012), banning of single stick sales (2017), introduction of mandatory graphic health warnings (2018) and tax stamps on tobacco products (2018) and more recently the ratification of the Protocol in October 2021 (5). Despite this progress, cigarettes continue to remain cheap and affordable in Ghana (1). For instance, the price of a pack of the most commonly sold brand of cigarette in Ghana is less than one USD. Although, Ghana does not have an active tobacco industry (British American Tobacco (BAT) ceased its local production in 2006), BAT continues to dominate sales of cigarettes and remains the dominant importer of cigarettes into the country via its manufacturing sites in Ibadan and Zaria in Nigeria (5). The distribution networks of Ghana’s leading tobacco companies are well organised in Ghana’s major urban cities including Greater Accra, Takoradi, Kumasi, and Tamale. Tobacco products including cigarettes in Ghana is mostly sold at unlicensed and unregulated points of sale such as traditional grocery retailers (also known as convenience or provision stores), street vendors, kiosks and drinking bars (6). An important challenge that exists in many African countries, including Ghana, is that most governments do not measure the size of illicit tobacco market nor analyze its features on a regular basis. To fully benefit from the Protocol, policymakers seek to

For peer review only

connect its normative guidance with empirical data and analysis on countries' illicit tobacco trade (ref). In light of the tobacco industry's use of illicit trade to oppose tobacco control measures such as tax increases (7), it is important to understand the scope and nature of the illicit tobacco trade. To date, there have been no scientific studies to estimate the size of the illicit cigarette market in Ghana (1). The only available estimates are those produced by the Euromonitor that reports an illicit cigarette market accounting for 39% of total cigarette sales in 2018 (up from 35% in 2017) (8). Estimates by Euromonitor have been criticized for being unreliable and inconsistent, and for lacking independence due to Euromonitor entering into business contracts with Philip Morris International (PMI) (5,11). The objectives of this study were to measure the extent of the illicit cigarette market in selected border and non-border towns in Ghana using an empty pack survey method from single stick sales. The study also assessed the nature and types of illicit cigarettes present in Ghana including the factors associated with illicit cigarettes sales in Ghana.

METHODS

Study sites

A cross sectional study was conducted during the months of August 2020 to January 2021 in five major cities in Ghana (Accra, Tamale, Kumasi, Takoradi and Bolgatanga) and three border towns (Aflao, Paga/Hamele and Elubu) across the three zones of Ghana (Northern, Middle and Coastal) (Figure 1). These districts were selected to represent socioeconomic, cultural and geographical diversity.

Insert figure 1

Research design

A modified approach based on the analysis of empty cigarette packs collected directly from retailors was used. This method was adapted from similar studies in India (9) and Bangladesh (10) and is particularly useful in countries where single stick sales are a

For peer review only

common practice. Within each large city or border town, ten smaller geographical areas were selected using Ghana Post Codes. A central point (such as a government building, market place or taxi station) was determined in each of them for retailor pack collection. A team of four research assistants and a coordinator walked 1 km along both sides of a busy street (0.5 km forward and 0.5 km back) starting from the central point to identify tobacco retailors. All retailers identified were provided with verbal and written information about the study and requested to sign a consent form if they agreed to participate. Following consent being obtained, an empty bag with a unique identifier was given to retailers and they were asked to deposit all cigarette packs emptied throughout the day as a result of single sticks of cigarette sales in the bag provided. The bags were collected back from the retailors at the end of a 24-hour period and retailors were given a small monetary incentive (up to a maximum amount of USD 10). Consenting retailors also participated in a 20-25 minutes survey on illicit cigarette sales, common brands, and pricing of cigarettes sold each day. Pack prices were recorded for each of the 10 and 20 stick packs. The sample size equation to obtain the minimum number of packs collected from each selected city/town was adapted from a toolkit for measuring illicit tobacco in LMICs (11). We obtained a minimum sample size of 2600 packs, assuming prevalence of illicit cigarette sales of 25%, with 95% level of confidence and margin of error of 0.15.

Classification of packs

Empty cigarette packs were cleaned and assigned unique IDs, and were analysed and their characteristics recorded. Pack data included the brand name, country of origin, the presence of graphical and/or textual health warnings, the language of the warning, the pack size (10/20 stick pack), and compliance of these warning messages with existing packaging requirement for Ghana. A conservative definition to classify an illicit

For peer review only

160 cigarette pack in Ghana according the Food and Drugs Authority (FDA), the regulatory body and the focal point for tobacco control in Ghana (12), includes at least one of the following attributes:

(a) Absence of authentic tax stamps;

(b) Absence of textual and pictorial warnings (Current pack warnings in Ghana are required to be a combined picture and text health warning in English to cover 50% of the front principal display area and 60% of the back principal display area of the pack, positioned in the lower portion) (13).

(c) Absence of the inscription "*FOR SALE IN GHANA ONLY*" displayed on the side panel of the product pack and

(d) Health warnings not in English

Trained research assistants evaluated tax stamp authenticity using the tax stamp mobile application developed by the Ghana Revenue Service (14).

Analysis

Data were first entered into excel, cleaned and analyzed via R studio version 1.4.1717. There was missing information from three of the pack data and these were removed from the final analysis. The unit of analysis was each cigarette pack. Continuous variables such as price/pack were changed to categorical (low and high price category) for 2-7 GHC and 8-14 GHC respectively (1USD=6GHC) for measures of association and continuous for the regression analysis. Descriptive information was reported as frequencies and percentages for city, country zone (northern, middle and coastal zones), retail shop type (drinking bars, convenience stores and kiosks), border and non-border towns, country of origin (based on the inscription on the packs on sale restricted to respective country eg. for sale in Togo only or Nigeria etc.) and illicit and licit cigarette. Pack characteristics such as pictorial health warning (absent/present), textual health warning (absent/present), warning labels in English (absent/present), tax stamps

For peer review only

(absent/present) and "*for sale in Ghana*" sign (absent/present) were captured. The relationship between illicit tobacco and the categorical variables (city type, country zone, type of shops, border and non-border town, price/packs, cigarette brand and country of origin) were first studied using $\chi 2$ or Fisher's exact test (when the number in the table was <6). Due to the binary nature of the outcome variable (licit/illicit), simple and multiple logistic regression was performed to evaluate the unadjusted and adjusted predictive values of the potential confounding variables respectively based on existing literature (15,16) (Figure 2). The results are presented as odds ratios (OR) with a 95% confidence interval, with significance set at an alpha level of 5% ($p \leq 0.05$).

Insert Figure 2

Patient and public Involvement

No patients and/or the public were not involved in the design, or conduct, or reporting, or dissemination plans of this research.

RESULTS

A total of 425 retailors were approached for the study, of whom 384 (90%) consented to collect packs and participate in the survey. An average of 12 cigarette packs were collected from single stick sales in a 24-hour period. A total of 4461 packs were collected from 384 retailors in the selected cities and towns. All retailors (100%) in the study sold single sticks. A total of 871 out of 4461 (20%, 95% CI: 18.34-20.66) packs were classified as illicit based on the criteria for classification approved by the FDA. A third (31%) of the packs collected from the northern zone of Ghana were illicit and almost seven out of 10 (69%) packs from the border towns were illicit. Almost all the packs collected from Aflao (Ghana-Togo border) were illicit (99%), followed by Tamale (46%) and the Paga/Hamele (Ghana-Burkina Faso border) (27%) and Elubu (21%) (Ghana - Cote d'Ivoire border) (Table 1). In terms of the retail selling points, three out of 10 (29%) packs collected from convenience stores were illicit, followed by

For peer review only

drinking bars (18%) (p<0.001). Over 60% of the packs collected within the price category of 2-7 GHC were illicit. The most common brand of cigarettes sold in Ghana is Rothmans Kingsize, London Brown/White and Pall Mall (Figure 3).

Insert Figure 3

Of all the 871 illicit packs collected, the most common brands of single stick sales were from Business Royal (24%), followed by Fine (21%) and Oris (12%).

Insert Figure 2

All packs from 555 and London Brown/White (manufactured by BAT) were licit (100%) (Table 1).

For peer review only

Table 1: **Determinants of illicit cigarette sale in Ghana**

	**Illicit cigarette packs (n=871)**	**Licit cigarettes packs (n=3590)**	**Total**
Country Zone			
Northern	368 (30.6)	835 (69.4)	1203 (100)
Middle	8 (1.2)	656 (98.8)	664 (100)
Coastal (south)	495 (19.1)	2099 (80.9)	2594 (100)
*P-value**	*<0.001*		
Border/non-border			

Border	493 (68.5)	227 (31.5)	720 (100)
Non-border	378 (10.1)	3363 (89.1)	3741 (100)
*P-value**	*<0.001*		
City/town (border/non border)			
Accra (non border)	17 (1.5)	1147 (98.5)	1164 (100)
Kumasi (non border)	8 (1.2)	651 (98.8)	659 (100)
Takoradi (non border)	1 (0.1)	767 (99.9)	768 (100)
Bolgatanga (non border)	7 (1.8)	390 (98.2)	397 (100)
Tamale (non border)	345 (45.8)	408 (54.2)	753 (100)
Elubu (Cote d'ivoire border)	44 (21.1)	165 (78.9)	209 (100)
Paga/Hamele (Burkina Faso border)	16 (26.6)	42 (72.4)	58 (100)
Aflao (Togo border)	433 (98.6)	20 (1.4)	453 (100)
P-value*	<0.001		
Shop type			
Drinking bar	477 (18.2)	2139 (81.8)	2616 (100)
Kiosks	31 (5.2)	563 (94.8)	594 (100)
Convenience stores	363 (29.0)	888 (71.0)	1251 (100)
*P-value**	*<0.001*		
Price/pack (GHC)			
Low price (2-7)	778 (61.2)	494 (38.8)	1272 (100)
High price (8-14)	93 (2.9)	3096 (97.1)	3189 (100)
P-value*	<0.001		
Cigarette brand (manufacturer)			
555 (BAT)	0 (0)	190 (100)	190(100)
London Brown/White (BAT)	0 (0)	928 (100)	928 (100)
Pallmall (BAT)	70 (14.2)	433 (85.8)	494 (100)
Business Royal (Independent Tobacco Inc)	210 (70.0)	90 (30.0)	300 (100)
Fine (unknown)	181 (78.3)	50 (21.6)	231 (100)
Rothmans Kingsize (BAT)	29 (1.6)	1798 (98.4)	1827 (100)
Oris (Oriental General Trading Inc)	107 (81.1)	35 (18.9)	132 (100)
Rothmans Royals (BAT)	99 (86.1)	20 (13.9)	115 (100)
Gold Seal (China Tobacco)	85 (91.4)	8 (8.6)	93 (100)
Tusker (BAT)	29 (100)	0 (0)	29 (100)
Others (Fisher, menthol, Cherry etc.)	61(50.0)	61 (50.0)	122 (100)
*P-value**	*<0.001*		

*P-value based on $\chi 2$ or Fisher's exact test

For the classification of illicit packs, majority were characterized by absence of tax stamps (94%), '*for sale in Ghana'* sign (92%), warning labels not in in English (77%) and absence of text and pictorial warning labels (28%).

Almost all the packs collected were the 20-stick pack (98%). The average price/pack of the 20-stick packs was 8.5 GHC and that for 10-stick was 3.3 GHC. Illicit packs had an average price/pack of 5.4 GHC (SD 1.5, range 2-12 GHC) whilst licit pack was 9.1 GHC (SD 2.1, range 2-14 GHC). Close to half of the illicit packs originated from Togo (51%), followed by Nigeria (15%) and then Cote d'Ivoire (10 %). About

For peer review only

2% of packs that were destined for Ghana were classified as illicit as the packs did not conform to the current labeling requirements as approved by FDA.

Table 2 shows the results from adjusted and unadjusted logistic regression of the factors associated with illicit cigarette sales in Ghana. The odds of illicit cigarette sales were 1.8 folds and 3.5 folds higher in convenience stores as compared to drinking bars in the unadjusted and adjusted models respectively (Table 2). Also, the sale of illicit cigarettes was 19.3 and 67.2 odds higher in border towns as compared to non-border towns in both the adjusted and unadjusted models respectively. The middle and coastal country zones had lower odds of illicit cigarettes sales than the northern zones in both the unadjusted and adjusted regression models respectively. Also, for every unit increase in price/pack, the odds of illicit cigarette consumption reduce by almost 60%.

For peer review only

Table 2: Unadjusted and unadjusted factors for illicit cigarette sales in Ghana

Variable	Unadjusted		Adjusted	
	OR	95% CI	OR	95% CI
Retail shop type				
Drinking bars	1		1	
Kiosks	0.25	0.17 - 0.35	0.52	0.28-0.96
Convenience stores	1.83	1.57-2.15	3.47	1.92-6.26
Country Zone				
Northern	1		1	
Middle	0.03	0.01-0.05	0.42	0.16 -1.08
Coastal	0.54	0.46-0.63	0.70	0.39-1.25
Border/non border towns				

Non-border town	1		1	
Border town	19.3	16.0-23.4	67.2	(44.2-102.2)
Pack price	0.39 (coef= -0.94	(0.37-0.42) (-0.99 to -0.88)	0.39 (coef=-0.95)	(0.36 -0.42) (-1.03 to -0.88)

DISCUSSION

This study found out that close to 20% of the packs collected were illicit of the total 4461 packs. Majority of the illicit packs were reported from Aflao (Ghana-Togo border) (99%) and Tamale (46%). Tamale, although not a border town, is the capital of the Northern region of Ghana, and has most of the cigarettes smuggled from Burkina Faso (8). Close to half of the illicit packs originated from Togo (51%), followed by Nigeria (15%) and then Cote d'Ivoire (10%). The most common brand of cigarettes sold in Ghana was from BAT including Rothmans Kingsize, London Brown/White and Pall Mall. One out four of the illicit packs belonged to Business Royal (Independent Tobacco Company), a fifth were from Fine (unknown company) and about one out of ten were from Oris brand (Oriental General Trading). The most common features identified for classifying packs as illicit were the absence of tax stamps, '*for sale in Ghana'* sign and warning labels not in English. Adjusted and unadjusted logistic regression models indicated that convenience stores, border towns, northern zone of the country and price/pack had higher odds of illicit cigarettes consumption for single stick sales in Ghana.

Our study provides an objective measure and describes the nature of the illicit cigarette market. This plays a critical role in developing comprehensive and effective tobacco control policies, particularly in countries within sub-Saharan Africa such as

For peer review only

Ghana, where data on illicit cigarettes sales is lacking. Our illicit cigarette estimates from single stick sales of 20%, is however, lower than the estimates of the Euromonitor (37% in 2018) (17), which is the only available estimate on illicit cigarettes market in Ghana. Nevertheless, the Euromonitor data is critisicied for lack of transparency and their and their funding source from the tobacco industry (TI) (17). The TI is known for quoting high estimates of the illicit market as a means of deterring governments from imposing tobacco tax increases, which contributes to ineffective tobacco control and lost opportunities for the governments to collect more revenue.

There are various methods to assess the extent of illicit tobacco in any country, such as measuring the difference between consumption and tax paid sales (gap analysis), interviewing smokers, examination of littered cigarette packs and econometric modeling (18). We employed a methodology particularly suitable in countries with single stick sales, similar to methods used in India (9), Pakistan (19), Bangladesh (10) and Argentina (20). Despite a ban on single stick sales, all retailors (100%) sold single sticks, calling for enforcement of the ban. Our estimates of illicit cigarette sales (20%) are also similar to countries with a higher tobacco use prevalence such as Pakistan (18%) and Argentina (14%) that used a similar methodology (19,20). Despite the lack of estimates of illicit cigarettes from many countries in the African Region, countries such as South Africa, Kenya, The Gambia and Nigeria have available estimates of their illicit market using different methods of estimation. Our estimates were found to be lower than South Africa (with over 30% of the total market being illicit) (21), Nigeria (26%) (22) and Kenya (26%) (23) but higher than the Gambia (8.6%) (24). With the recent ratification of the Protocol in Ghana, and estimates suggesting one out of five cigarette packs to be illicit, there is an urgent

For peer review only

301 need for governments to address this by fully implementing ratified protocol (which
302 has specific requirements to improve traceability of tobacco products and increase
303 tobacco industry accountability).
304 British American Tobacco (BAT) continues to dominate sales of cigarettes as
305 evidenced by the most common cigarettes sold in Ghana (Rothmans Kingsize,
306 London Brown/White and Pall Mall). This is largely due to the company's long
307 history in Ghana (25). While the company ceased domestic production in 2006, it
308 remains the dominant importer of cigarettes into the country (25). There are also very
309 low-priced brands available, such as BAT's Tusker brand (of which all packs were
310 illicit). While, all packs from London Brown/White were found to be licit, about 14%
311 of Pall Mall and 1.6% of Rothmans Kingsize were illicit, demonstrating the
312 possibility of the industry's involvement in illicit trade. [Note: Removed by editor at
313 acceptance. Please see final version of manuscript.] Further, the small-scale convenience
314 stores were found to be a major selling point of illicit cigarettes. These are legally
315 operating, widely available settings to the low-income Ghanaian smoker (who prefers
316 to buy single stick) widely available in both rural and urban locations. Convenience
317 stores were also found to have higher odds of illicit cigarette consumption as
318 compared to drinking bars in both the adjusted and unadjusted logistic regression
319 models, indicating that it may be an important predictor of illicit cigarette sales in the
320 country.
321 Geography was found to play an important role in the illicit cigarette market in
322 Ghana. A third of the packs collected from the northern zone of the country were
323 found to be illicit. According to the Euromonitor (8), the north of Ghana sees
324 particularly strong illicit trade, with most smuggling from Burkina Faso finding their
325 way to this region into Tamale (17). This could also be linked to the high smoking

For peer review only

prevalence and lower income population in the region as compared to other regions (27). Similarly, border towns were also found to be strong predictors of illicit cigarette sales. Six out of 10 packs collected from border towns were illicit and almost 100% of the packs collected from Aflao (Ghana-Togo border), and close to half of the packs from Tamale (large city in Northern Ghana linked to Burkina Faso) were found to be illicit. Border towns have been found to be more vulnerable to the trade of illicit cigarette and tobacco products in Vietnam (28) and Georgia (29). Our findings reinforces the need for strengthening patrolling and border control in addition to building capacity and training for authorities belonging to customs, police and immigration. The illicit cigarettes originated from Togo (51%), followed by Nigeria (15%) and then Cote d'Ivoire (10%). [Note: Removed by editor at acceptance. Please see final version of manuscript.]

In terms of pricing of cigarettes, illicit packs were found to be almost 50% cheaper than licit packs. Africa in general, lags behind other regions (such as European and the Americas) in implementing strong tobacco tax policies (1). Close to 90% of the illicit packs were belonged to the low price category (2-7 GHC). Currently, the total excise tax on tobacco products in Ghana, accounts for only 31.8% of the average retail price (30). Also, over half of the smuggled cigarettes in the study originated from Togo where a pack of cigarettes is priced at about one USD and is about 0.50 USD in Ghana (30). The link between tobacco taxation and smuggling has been doubtful and inconsistent (31). According to a report by the World Bank (32), taxes and prices have only a limited impact on illicit cigarette market share at country level, contrary to arguments by the tobacco industry. The African region, with low prices and low taxation on tobacco products and high levels of smuggling, provides a good

For peer review only

illustration of this observation. This calls for more research to understand the relationship between tobacco taxation and smuggling in Africa.

Our study findings should be considered in the light of some limitations. First, despite the wide geographical dispersion in the three zones of the country (northern, middle and coastal), the representativeness to the country is limited. Also, as data was collected during COVID-19 lockdown period in Ghana and we could not explore other border towns that were planned due to pertaining restrictions at that time. Secondly, the empty pack collection relies on retailers to provide us with all the empty packs from previous day's single stick sales. It could be possible that some retailers would want to hide the illegal packs, which could underestimate our findings. Nevertheless, retailors were motivated with a monetary incentive, which, to an extent, mitigated this issue.

CONCLUSION

Our study found a total of 20% illicit packs in the entire sample of packs collected across the eight border and non-border towns/cities in Ghana. This study provides valuable information for policymakers and law enforcement in the region and bringing to light the inadequacy of the current monitoring and regulatory activities of the FDA and customs. Our findings have two important policy implications; first, the regulatory body and the focal point for tobacco control in Ghana (FDA) in collaboration with the customs, police and immigration, should strengthen the supply chain control and market surveillance at retail points in the towns and cities, particularly those close to the Ghana-Togo and Ghana-Burkina Faso border in the northern and coastal zones of the country, aside from border monitoring and transportation tracing. Secondly, with the introduction of Tax Stamp Policy since March 2018, Ghana should also consider the implementation of a supply chain control

For peer review only

that resembles a track and trace system (like Kenya), independent of any industry influence to effectively monitor the illicit market.

ACKNOWLEDGEMENTS

We would also like to thank Michael Ababio and Christopher Bekoe for their role and contribution in pack collection and fieldwork. We would also like to thank all the retailors that provided information and packs for this survey.

CONTRIBUTORS

AS, FD, AG, TK, HR and EOD contributed to the design, conception, acquisition, analysis and interpretation of the project and data; the drafting and revision of the manuscript and the approval of the final version to be published. AS and DL contributed to the acquisition of data. LB contributed to the design and conception of the project. OB and AG contributed to the drafting and revision of the manuscript and the approval of the final version to be published.

COMPETING INTEREST

None declared

FUNDING

This work was supported by the Medical Research Council [grant number MR/P027946/2] with funding from the Global Challenges Research Fund and with additional funding from the University of Edinburgh's Scottish Funding Council Global Challenges Research Fund (GCRF) allocation.

Ethics Approval

The study protocol was approved by the Committee on Human Research, Publication and Ethics (Reference number: CHRPE/AP/441/18) and the University of Bath's Research Ethics Approval Committee for Health (REACH) (EP 19/20 063).

For peer review only

For peer review only - http://bmjopen.bmj.com/site/about/guidelines.xhtml

Data sharing statement

The data are owned and shared by the Tobacco Control Capacity Program (TCCP) and the School of Public Health, KNUST, Ghana. Requests for data sharing can be made to artisingh_uk@yahoo.com/arti.singh@tuni.fi

Legends for figures

Figure 1: Location of the eight cities for pack collection in Ghana (black arrows)

Figure 2: Causal diagram of illicit cigarette consumption from single stick sales in Ghana (potential confounders were border towns, country zone, pack prices and type of retail shop)

Figure 3: Cigarette brands sold in Ghana

REFERENCES

1. WHO. Who Report on the Global Tobacco Epidemic, 2021. 2021;

2. Confronting illicit tobacco trade: a global review of country experiences.

3. Joossens L, Merriman D, Ross H et al IU against T and L. How eliminating the global illicit cigarette trade would increase tax revenue and save lives. 2009 [cited 2022 Feb 3];30. Available from: https://theunion.org/technical-publications/how-eliminating-the-global-illicit-cigarette-trade-would-increase-tax-revenue-and-save-lives

4. Transnational Alliance to Combat Illicit Trade. Mapping the Impact of Illicit Trade on the Sustainable Development Goals. 2019; Available from: www.tracit.org/publications.html

5. Singh A, Owusu-Dabo E, Mdege N, McNeill A, Britton J, Bauld L. A situational analysis of tobacco control in Ghana: progress, opportunities and challenges. Journal of Global Health Reports. 2020;

6. Tobacco in Ghana | Market Research Report | Euromonitor [Internet]. [cited

For peer review only

2021 Aug 17]. Available from: https://www.euromonitor.com/tobacco-in-ghana/report

7. Joossens L, Merriman D, Ross H, Raw M. The impact of eliminating the global illicit cigarette trade on health and revenue. Addiction. 2010 Sep 1;105(9):1640–9.

8. ICTD. Tobacco in Ghana | Market Research Report | Euromonitor [Internet]. [cited 2022 Jul 30]. Available from: https://www.euromonitor.com/tobacco-in-ghana/report

9. John RM, Ross H. Illicit cigarette sales in Indian cities: findings from a retail survey. Tob Control. 2018 Nov 1;27(6):684–8.

10. Abdullah SM, Huque R, Bauld L, Ross H, Gilmore A, John RM, et al. Estimating the Magnitude of Illicit Cigarette Trade in Bangladesh: Protocol for a Mixed-Methods Study. International Journal of Environmental Research and Public Health 2020, Vol 17, Page 4791 [Internet]. 2020 Jul 3 [cited 2022 Jan 5];17(13):4791. Available from: https://www.mdpi.com/1660-4601/17/13/4791/htm

11. Michal S, Guillermo P, Blecher E. A Toolkit on Measuring Illicit Trade in Tobacco Products. 2020;

12. Welcome to Ghana Food And Drugs Authority | Home [Internet]. [cited 2022 Jan 5]. Available from: http://www.fdaghana.gov.gh/index.php

13. Campaign for Tobacco-Free Kids. Ghana Details | Tobacco Control Laws [Internet]. 2022 [cited 2022 Jul 30]. Available from: https://www.tobaccocontrollaws.org/legislation/country/ghana/pl-health-warnings

14. GRA introduces Ghana Tax Stamp Authenticator mobile app [Internet]. [cited

For peer review only

2022 Feb 23]. Available from:
https://www.ghanaweb.com/GhanaHomePage/business/GRA-introduces-Ghana-Tax-Stamp-Authenticator-mobile-app-771674

15. Vellios N, Van Walbeek C, Ross H. Measuring the illicit cigarette market in the absence of pack security features: A case study of South Africa. Tobacco Control. 2021 Feb 25;

16. John RM, Ross H. Consumption of illicit cigarettes in India: findings from a retail survey. Tobacco Induced Diseases. 2018;

17. Profile I. Tobacco in. 2011;(October):1–32.

18. A Toolkit on Measuring Illicit Trade in Tobacco Products | Tobacconomics [Internet]. [cited 2022 Jan 5]. Available from: https://tobacconomics.org/research/a-toolkit-on-measuring-illicit-trade-in-tobacco-products/

19. Khan A, Dobbie F, Siddiqi K, Ansaari S, Abdullah SM, Iqbal R, et al. Illicit cigarette trade in the cities of Pakistan: comparing findings between the consumer and waste recycle store surveys. Tobacco Control [Internet]. 2021 Apr 15 [cited 2021 Dec 30];1–7. Available from: https://tobaccocontrol.bmj.com/content/early/2021/04/14/tobaccocontrol-2020-056386

20. Pizarro ME, Giacobone G, Shammah C, Stoklosa M. Illicit tobacco trade: empty pack survey in eight Argentinean cities. Tobacco Control [Internet]. 2021 Apr 8 [cited 2021 Dec 30];0:1–7. Available from: https://tobaccocontrol.bmj.com/content/early/2021/04/07/tobaccocontrol-2020-056405

21. Vellios N, van Walbeek C, Ross H. Illicit cigarette trade in South Africa:

For peer review only

2002–2017. Tobacco Control [Internet]. 2020 Oct 1 [cited 2021 Dec 30];29(Suppl 4):s234–42. Available from: https://tobaccocontrol.bmj.com/content/29/Suppl_4/s234

22. Adeniran AP, Ekeruche MA, Castradori M. Illicit Tobacco in Nigeria [Internet]. Centre for the Study of the Economies of Africa (CSEA); 2020 [cited 2021 Dec 23]. Available from: https://www.africaportal.org/publications/illicit-tobacco-nigeria/

23. Ross H. Tracking and tracing tobacco products in Kenya. Preventive Medicine. 2017 Dec 1;105:S15–8.

24. Chisha Z, Janneh ML, Ross H. Consumption of legal and illegal cigarettes in the Gambia. Tob Control [Internet]. 2020;29:254–9. Available from: http://tobaccocontrol.bmj.com/

25. Owusu-Dabo E, Lewis S, McNeill A, Anderson S, Gilmore A, Britton J. Smoking in Ghana: A review of tobacco industry activity. Tobacco Control. 2009;

26. Morris P. British American Tobacco Fights Dirty In West Africa British American Tobacco Fights Dirty In West Africa. 2021;

27. Yawson AE, Baddoo A, Hagan-Seneadza NA, Calys-Tagoe B, Hewlett S, Dako-Gyeke P, et al. Tobacco use in older adults in Ghana: Sociodemographic characteristics, health risks and subjective wellbeing. BMC Public Health. 2013;

28. Nguyen HTT, Giang LT, Pham TN. Empirical analysis on the illicit trade of cigarettes in Vietnam. Tobacco Control [Internet]. 2020 Oct 1 [cited 2021 Dec 30];29(Suppl 4):s281–6. Available from: https://tobaccocontrol.bmj.com/content/29/Suppl_4/s281

29. Little M, Ross H, Bakhturidze G, Kachkachishvili I. Illicit tobacco trade in Georgia: prevalence and perceptions. Tobacco Control [Internet]. 2020 Oct 1 [cited 2021 Dec 30];29(Suppl 4):s227–33. Available from: https://tobaccocontrol.bmj.com/content/29/Suppl_4/s227

30. HealthBridge. Tobacco Taxation Factsheet [Internet]. 2020. Available from: https://healthbridge.ca/images/uploads/library/Factsheet_2_-_Taxation_final.pdf

31. Economic WA. Tobacco Taxation Factsheet : Smoking in Mali : The Facts Tobacco Taxation in Mali. 2019;2019–20.

32. World Bank. Confronting Llicit Tobacco Trade. 2019; Available from: https://thedocs.worldbank.org/en/doc/662791548435048907-0090022019/original/WBGTobaccoIllicitTradeSACU.pdf

For peer review only

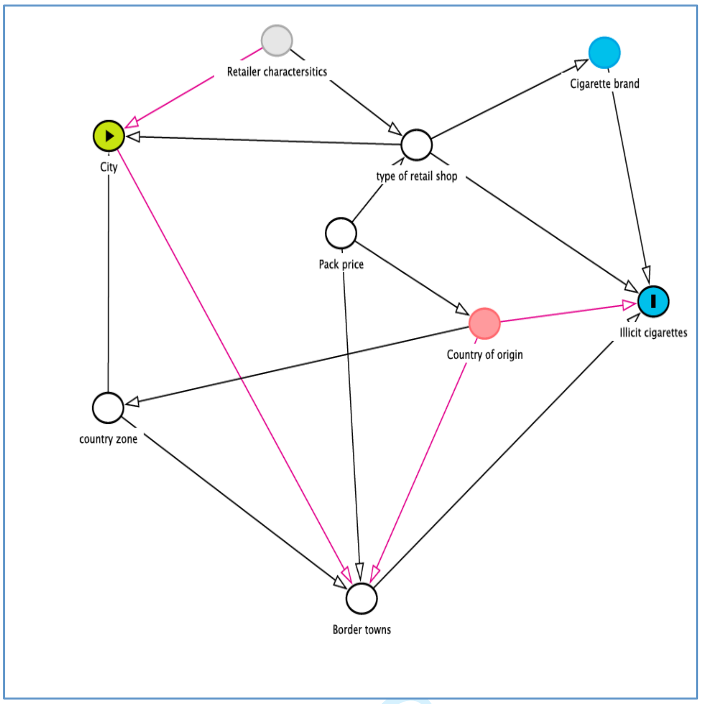

Figure 2: Causal diagram of illicit cigarette consumption from single stick sales in Ghana (potential confounders were border towns, country zone, pack prices and type of retail shop)

For peer review only

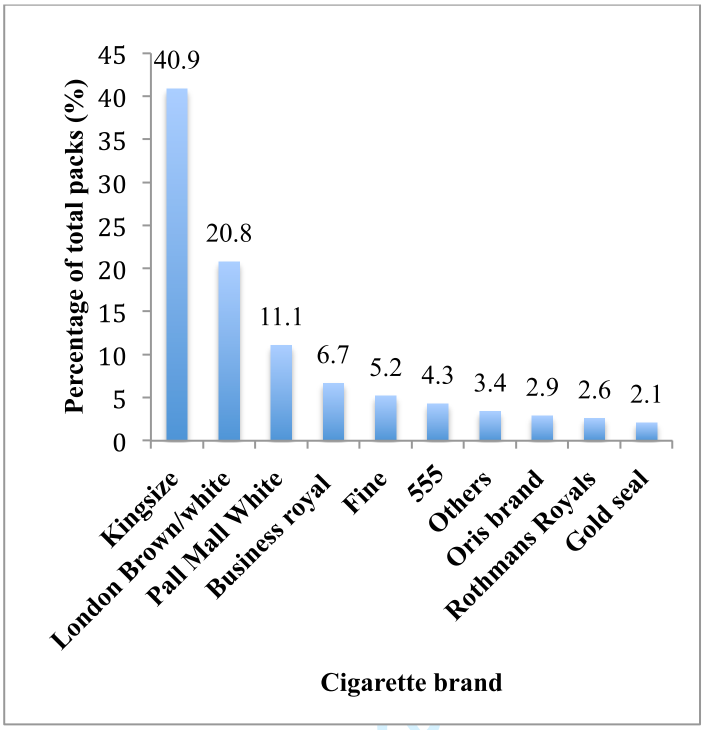

Figure 3: Common cigarette brands sold in Ghana

For peer review only

STROBE Statement—Checklist of items that should be included in reports of ***cross-sectional studies***

	Item No	Recommendation	Page No
**Title and abstract**	1	(*a*) Indicate the study's design with a commonly used term in the title or the abstract	Title and abstract
		(*b*) Provide in the abstract an informative and balanced summary of what was done and what was found	Abstract
**Introduction**			
Background/rationale	2	Explain the scientific background and rationale for the investigation being reported	115-138
Objectives	3	State specific objectives, including any prespecified hypotheses	135-138
**Methods**			
Study design	4	Present key elements of study design early in the paper	148-171
Setting	5	Describe the setting, locations, and relevant dates, including periods of recruitment, exposure, follow-up, and data collection	141-144
Participants	6	(*a*) Give the eligibility criteria, and the sources and methods of selection of participants	151-159
Variables	7	Clearly define all outcomes, exposures, predictors, potential confounders, and effect modifiers. Give diagnostic criteria, if applicable	243-249
Data sources/ measurement	8*	For each variable of interest, give sources of data and details of methods of assessment (measurement). Describe comparability of assessment methods if there is more than one group	196-202
Bias	9	Describe any efforts to address potential sources of bias	252-257
Study size	10	Explain how the study size was arrived at	168-171
Quantitative variables	11	Explain how quantitative variables were handled in the analyses. If applicable, describe which groupings were chosen and why	194-196
Statistical methods	12	(*a*) Describe all statistical methods, including those used to control for confounding	202-210
		(*b*) Describe any methods used to examine subgroups and interactions	N/A
		(*c*) Explain how missing data were addressed	238-239
		(*d*) If applicable, describe analytical methods taking account of sampling strategy	N/A
		(*e*) Describe any sensitivity analyses	N/A
**Results**			
Participants	13*	(a) Report numbers of individuals at each stage of study—eg numbers potentially eligible, examined for eligibility, confirmed eligible, included in the study, completing follow-up, and analysed	214-217, Table 1
		(b) Give reasons for non-participation at each stage	N/A
		(c) Consider use of a flow diagram	N/A
Descriptive data	14*	(a) Give characteristics of study participants (eg demographic, clinical, social) and information on exposures and potential confounders	217-228
		(b) Indicate number of participants with missing data for each variable of interest	N/A
Outcome data	15*	Report numbers of outcome events or summary measures	302-306
Main results	16	(*a*) Give unadjusted estimates and, if applicable, confounder-adjusted estimates and their precision (eg, 95% confidence interval). Make clear	267-274, Table 2

For peer review only - http://bmjopen.bmj.com/site/about/guidelines.xhtml

		which confounders were adjusted for and why they were included	
		(*b*) Report category boundaries when continuous variables were categorized	241-243
		(*c*) If relevant, consider translating estimates of relative risk into absolute risk for a meaningful time period	N/A
Other analyses	17	Report other analyses done—eg analyses of subgroups and interactions, and sensitivity analyses	N/A
**Discussion**			
Key results	18	Summarise key results with reference to study objectives	282-302
Limitations	19	Discuss limitations of the study, taking into account sources of potential bias or imprecision. Discuss both direction and magnitude of any potential bias	399-411
Interpretation	20	Give a cautious overall interpretation of results considering objectives, limitations, multiplicity of analyses, results from similar studies, and other relevant evidence	303-398
Generalisability	21	Discuss the generalisability (external validity) of the study results	399-401
**Other information**			
Funding	22	Give the source of funding and the role of the funders for the present study and, if applicable, for the original study on which the present article is based	458-461

*Give information separately for exposed and unexposed groups.

Note: An Explanation and Elaboration article discusses each checklist item and gives methodological background and published examples of transparent reporting. The STROBE checklist is best used in conjunction with this article (freely available on the Web sites of PLoS Medicine at http://www.plosmedicine.org/, Annals of Internal Medicine at http://www.annals.org/, and Epidemiology at http://www.epidem.com/). Information on the STROBE Initiative is available at www.strobe-statement.org.

For peer review only

BMJ Open

Extent of illicit cigarette market from Single Stick Sales in Ghana: findings from a cross sectional survey

Journal:	*BMJ Open*
Manuscript ID	bmjopen-2022-062476.R2
Article Type:	Original research
Date Submitted by the Author:	30-Oct-2022
Complete List of Authors:	Singh, Arti; Tampere University<br>Ross, Hana; University of Cape Town Research Unit on the Economics of Excisable Products<br>Dobbie, Fiona; The University of Edinburgh College of Medicine and Veterinary Medicine, ;<br>Gallagher, Allen; University of Bath<br>Kinnunen, Tarja; Tampere University<br>Logo, Divine; Ghana Health Service, Research & Development Division-Ghana Health Service; Ministry of Health/Ghana health Service<br>Boateng, Olivia A.; Food and Drug Authority, Tobacco Control and Substance Abuse<br>Gilmore, Anna; University of Bath, Department for Health<br>Bauld, Linda; University of Edinburgh, Usher Institute and UK Centre for Tobacco and Alcohol Studies, College of Medicine and Veterinary Medicine<br>Owusu-Dabo, Ellis; Kwame Nkrumah University of Science and Technology, Department of Global Health
**Primary Subject Heading**:	Public health
Secondary Subject Heading:	Epidemiology, Global health, Public health, Smoking and tobacco
Keywords:	PUBLIC HEALTH, Epidemiology < TROPICAL MEDICINE, STATISTICS & RESEARCH METHODS

SCHOLARONE™
Manuscripts

I, the Submitting Author has the right to grant and does grant on behalf of all authors of the Work (as defined in the below author licence), an exclusive licence and/or a non-exclusive licence for contributions from authors who are: i) UK Crown employees; ii) where BMJ has agreed a CC-BY licence shall apply, and/or iii) in accordance with the terms applicable for US Federal Government officers or employees acting as part of their official duties; on a worldwide, perpetual, irrevocable, royalty-free basis to BMJ Publishing Group Ltd ("BMJ") its licensees and where the relevant Journal is co-owned by BMJ to the co-owners of the Journal, to publish the Work in this journal and any other BMJ products and to exploit all rights, as set out in our licence.

The Submitting Author accepts and understands that any supply made under these terms is made by BMJ to the Submitting Author unless you are acting as an employee on behalf of your employer or a postgraduate student of an affiliated institution which is paying any applicable article publishing charge ("APC") for Open Access articles. Where the Submitting Author wishes to make the Work available on an Open Access basis (and intends to pay the relevant APC), the terms of reuse of such Open Access shall be governed by a Creative Commons licence – details of these licences and which Creative Commons licence will apply to this Work are set out in our licence referred to above.

Other than as permitted in any relevant BMJ Author's Self Archiving Policies, I confirm this Work has not been accepted for publication elsewhere, is not being considered for publication elsewhere and does not duplicate material already published. I confirm all authors consent to publication of this Work and authorise the granting of this licence.

For peer review only

Extent of illicit cigarette market from Single Stick Sales in Ghana: findings from a cross sectional survey

Arti Singh[1, 5], Hana Ross[2], Fiona Dobbie[3], Allen Gallagher[4], Tarja I Kinnunen[5], Divine D. Logo[1], Olivia A. Boateng[6], Anna Gilmore[4], Linda Bauld[3], Ellis Owusu-Dabo[1]

Corresponding author:

Arti Singh
School of Public Health
KNUST, Kumasi
Ghana
Email: artisingh_uk@yahoo.com/arti.singh@tuni.fi

Tel: (233)-244464576

Affiliations

[1]School of Public Health, Kumasi, Ghana

[2]University of Cape Town, South Africa

[3]University of Edinburgh, UK

[4]University of Bath, UK

[5]Tampere University, Finland

[6]Food and Drug Authority, Ghana

Keywords: Illicit, cigarette, Ghana, packs, survey, tobacco

For peer review only

ABSTRACT

Objective This study aims to measure the extent of illicit cigarette consumption from single stick sales, to determine the nature and types of illicit cigarettes present in Ghana, and to identify the factors associated with illicit cigarettes consumption in Ghana.

Design A Cross-sectional study using empty cigarette packs generated by 1 day's single stick cigarette sales collected from cigarette vendors.

Setting Five large cities (Accra, Kumasi, Takoradi, Tamale, and Bolgatanga) and three border towns (Aflao, Paga/Hamele and Elubo) in the northern, middle and coastal belt of Ghana.

Procedure and Participants Ten areas were randomly selected in each city/town, and all shops selling cigarettes within 1 km of the central point were surveyed.

Outcome measures (1) estimates of the share of illicit cigarette packs in the total cigarette sales from vendors selling single stick cigarettes in Ghana; (2) nature and types of illicit cigarette packs; and (3) factors associated with illicit cigarette sales in Ghana.

Results

Of a total of 4461 packs, about 20% (95% CI: 18.3-20.7) were found to be illicit. Aflao (Ghana-Togo border) and Tamale (Ghana-Burkina Faso border) had the highest percentage of illicit cigarette sales at 99% and 46% respectively ($p<0.001$). Over half of the illicit packs originated from Togo (51%), followed by Nigeria (15%) and then Cote d'Ivoire (10%). Adjusted and unadjusted logistic regression models indicated that convenience stores, border towns, pack price and the northern zone had higher odds of illicit cigarette sales.

For peer review only

Conclusion To effectively tackle illicit cigarettes, market surveillance and strengthening supply chain control are required, particularly at the border towns and the northern region of the country.

Strengths and limitations of this study

- The empty pack survey required little time and resources to conduct.
- Some retailers may not provide all the packs over the last 24 hours, which could underestimate our findings.
- The study was limited to five cities and three border towns and is not representative of illicit cigarettes sales in Ghana as a whole

INTRODUCTION

Illicit tobacco trade continues to remain a threat to global tobacco control efforts. While tobacco consumption is decreasing globally, rapid population growth, increased advertising by the tobacco industry, and growing tobacco consumption among young people in Africa may result in increased number of smokers in the region (1). Further, the availability and accessibility of cheap, illicit tobacco products is particularly attractive to the region's most vulnerable young population and low-income smokers (2).

Illicit trade of tobacco products is a major public health problem as lower prices of illicit cigarettes lead to increased cigarette consumption (3). Despite the difficulties in measuring the extent of illicit tobacco in the market, available estimates indicates that it was about 11.6% worldwide in 2007 and almost 10% in 2015 (3), and these figures are higher for low and middle income countries (LMICs) including those in the African Region. In response to the threat posed by illicit tobacco trade, the WHO FCTC Protocol to Eliminate Illicit Trade in Tobacco Products (hereby referred to as "the

For peer review only

Protocol") entered into force in 2018 (4). This Protocol gives countries an opportunity to prevent tobacco-related morbidity and mortality by enhancing tobacco supply chain control. Countries that ratify the Protocol commit themselves to adopting a variety of measures, including track and trace systems to prevent and counter illicit trade.

Ghana, one of the first countries to ratify the World Health Organization's (WHO) Framework Convention on Tobacco Control (FCTC) in 2004, has made some significant progress in tobacco control such as introducing an early advertising ban (1982), the passage of the Tobacco Control Act (in 2012), banning of single stick sales (2017), introduction of mandatory graphic health warnings (2018) and tax stamps on tobacco products (2018) and more recently the ratification of the Protocol in October 2021 (5). Despite this progress, cigarettes continue to remain cheap and affordable in Ghana (1). For instance, the price of a pack of the most commonly sold brand of cigarette in Ghana is less than one USD. Although, Ghana does not have an active tobacco industry (British American Tobacco (BAT) ceased its local production in 2006), BAT continues to dominate sales of cigarettes and remains the dominant importer of cigarettes into the country via its manufacturing sites in Ibadan and Zaria in Nigeria (5). The distribution networks of Ghana's leading tobacco companies are well organised in Ghana's major urban cities including Greater Accra, Takoradi, Kumasi, and Tamale. Tobacco products including cigarettes in Ghana is mostly sold at unlicensed and unregulated points of sale such as traditional grocery retailers (also known as convenience or provision stores), street vendors, kiosks and drinking bars (6).

An important challenge that exists in many African countries, including Ghana, is that most governments do not measure the size of illicit tobacco market nor analyze its features on a regular basis. To fully benefit from the Protocol, policymakers seek to connect its normative guidance with empirical data and analysis on countries' illicit

For peer review only

tobacco trade. In light of the tobacco industry's use of illicit trade to oppose tobacco control measures such as tax increases (7), it is important to understand the scope and nature of the illicit tobacco trade. To date, there have been no scientific studies to estimate the size of the illicit cigarette market in Ghana (1). The only available estimates are those produced by the Euromonitor that reports an illicit cigarette market accounting for 39% of total cigarette sales in 2018 (up from 35% in 2017) (8). Estimates by Euromonitor have been criticized for being unreliable and inconsistent, and for lacking independence due to Euromonitor entering into business contracts with Philip Morris International (PMI) (5). The objectives of this study were to measure the extent of the illicit cigarette market in selected border and non-border towns in Ghana using an empty pack survey method from single stick sales. The study also assessed the nature and types of illicit cigarettes present in Ghana including the factors associated with illicit cigarettes sales in Ghana.

METHODS

Study sites

A cross sectional study was conducted during the months of August 2020 to January 2021 in five major cities in Ghana (Accra, Tamale, Kumasi, Takoradi and Bolgatanga) and three border towns (Aflao, Paga/Hamele and Elubu) across the three zones of Ghana (Northern, Middle and Coastal). These districts were selected to represent socioeconomic, cultural and geographical diversity.

Research design

A modified approach based on the analysis of empty cigarette packs collected directly from retailers was used. This method was adapted from similar studies in India (9) and Bangladesh (10) and is particularly useful in countries where single stick sales are a common practice. Within each large city or border town, ten smaller geographical areas were selected using Ghana Post Codes. A central point (such as a government building,

For peer review only

market place or taxi station) was determined in each of them for retailor pack collection. A team of four research assistants and a coordinator walked 1 km along both sides of a busy street (0.5 km forward and 0.5 km back) starting from the central point to identify tobacco retailers. All retailers identified were provided with verbal and written information about the study and requested to sign a consent form if they agreed to participate. Following consent being obtained, an empty bag with a unique identifier was given to retailers and they were asked to deposit all cigarette packs emptied throughout the day as a result of single sticks of cigarette sales in the bag provided. The bags were collected back from the retailers at the end of a 24-hour period and retailers were given a small monetary incentive (up to a maximum amount of USD 10). Consenting retailers also participated in a 20-25 minutes survey on illicit cigarette sales, common brands, and pricing of cigarettes sold each day. Pack prices were recorded for each of the 10 and 20 stick packs. The sample size equation to obtain the minimum number of packs collected from each selected city/town was adapted from a toolkit for measuring illicit tobacco in LMICs (11). We obtained a minimum sample size of 2600 packs, assuming prevalence of illicit cigarette sales of 25%, with 95% level of confidence and margin of error of 0.15.

Classification of packs

Empty cigarette packs were cleaned and assigned unique IDs, and were analysed and their characteristics recorded. Pack data included the brand name, country of origin, the presence of graphical and/or textual health warnings, the language of the warning, the pack size (10/20 stick pack), and compliance of these warning messages with existing packaging requirement for Ghana. A conservative definition to classify an illicit cigarette pack in Ghana according the Food and Drugs Authority (FDA), the regulatory

For peer review only

body and the focal point for tobacco control in Ghana (12), includes at least one of the following attributes:

(a) Absence of authentic tax stamps;

(b) Absence of textual and pictorial warnings (Current pack warnings in Ghana are required to be a combined picture and text health warning in English to cover 50% of the front principal display area and 60% of the back principal display area of the pack, positioned in the lower portion) (13).

(c) Absence of the inscription "*FOR SALE IN GHANA ONLY*" displayed on the side panel of the product pack and

(d) Health warnings not in English

Trained research assistants evaluated tax stamp authenticity using the tax stamp mobile application developed by the Ghana Revenue Service (14).

Analysis

Data were first entered into excel, cleaned and analyzed via R studio version 1.4.1717. There was missing information from three of the pack data and these were removed from the final analysis. The unit of analysis was each cigarette pack. Continuous variables such as price/pack were changed to categorical (low and high price category) for 2-7 GHC and 8-14 GHC respectively (1USD=6GHC) for measures of association and continuous for the regression analysis. Descriptive information was reported as frequencies and percentages for city, country zone (northern, middle and coastal zones), retail shop type (drinking bars, convenience stores and kiosks), border and non-border towns, country of origin (based on the inscription on the packs on sale restricted to respective country eg. for sale in Togo only or Nigeria etc.) and illicit and licit cigarette. Pack characteristics such as pictorial health warning (absent/present), textual health warning (absent/present), warning labels in English (absent/present), tax stamps (absent/present) and "*for sale in Ghana*" sign (absent/present) were captured. The

For peer review only

relationship between illicit tobacco and the categorical variables (city type, country zone, type of shops, border and non-border town, price/packs, cigarette brand and country of origin) were first studied using $\chi2$ or Fisher's exact test (when the number in the table was <6). Due to the binary nature of the outcome variable (licit/illicit), simple and multiple logistic regression was performed to evaluate the unadjusted and adjusted predictive values of the potential confounding variables respectively based on existing literature (15,16) (Figure 1). Subsequently, a cluster analysis was performed to identify the effect of vendors on the sale of illicit single e stick sales in Ghana. The results are presented as odds ratios (OR) with a 95% confidence interval, with significance set at an alpha level of 5% ($p \leq 0.05$).

Insert Figure 1

Patient and public Involvement

No patients and/or the public were involved in the design, or conduct, or reporting, or dissemination plans of this research.

RESULTS

A total of 425 retailers were approached for the study, of whom 384 (90%) consented to collect packs and participate in the survey. An average of 12 cigarette packs were collected from single stick sales in a 24-hour period. A total of 4461 packs were collected from 384 retailers in the selected cities and towns. All retailers (100%) in the study sold single sticks. A total of 871 out of 4461 (20%, 95% CI: 18.34-20.66) packs were classified as illicit based on the criteria for classification approved by the FDA. A third (31%) of the packs collected from the northern zone of Ghana were illicit and almost seven out of 10 (69%) packs from the border towns were illicit. Almost all the packs collected from Aflao (Ghana-Togo border) were illicit (99%), followed by Tamale (46%) and the Paga/Hamele (Ghana-Burkina Faso border) (27%) and Elubu (21%) (Ghana - Cote d'Ivoire border) (Table 1). In terms of the retail selling points,

For peer review only

three out of 10 (29%) packs collected from convenience stores were illicit, followed by drinking bars (18%) (p<0.001). Over 60% of the packs collected within the price category of 2-7 GHC were illicit. The most common brand of cigarettes sold in Ghana is Rothmans Kingsize, London Brown/White and Pall Mall (Figure 2).

Insert Figure 2

Of all the 871 illicit packs collected, the most common brands of single stick sales were from Business Royal (24%), followed by Fine (21%) and Oris (12%).

All packs from 555 and London Brown/White (manufactured by BAT) were licit (100%) (Table 1).

Table 1: **Determinants of illicit cigarette sale in Ghana**

	**Illicit cigarette packs (n=871)**	**Licit cigarettes packs (n=3590)**	**Total**
Country Zone			
Northern	368 (30.6)	835 (69.4)	1203 (100)
Middle	8 (1.2)	656 (98.8)	664 (100)
Coastal (south)	495 (19.1)	2099 (80.9)	2594 (100)
*P-value**		*<0.001*	
Border/non-border			
Border	493 (68.5)	227 (31.5)	720 (100)
Non-border	378 (10.1)	3363 (89.1)	3741 (100)
*P-value**		*<0.001*	
City/town (border/non border)			
Accra (non border)	17 (1.5)	1147 (98.5)	1164 (100)
Kumasi (non border)	8 (1.2)	651 (98.8)	659 (100)
Takoradi (non border)	1 (0.1)	767 (99.9)	768 (100)
Bolgatanga (non border)	7 (1.8)	390 (98.2)	397 (100)
Tamale (non border)	345 (45.8)	408 (54.2)	753 (100)
Elubu (Cote d'ivoire border)	44 (21.1)	165 (78.9)	209 (100)
Paga/Hamele (Burkina Faso border)	16 (26.6)	42 (72.4)	58 (100)
Aflao (Togo border)	433 (98.6)	20 (1.4)	453 (100)
P-value*		<0.001	
Shop type			
Drinking bar	477 (18.2)	2139 (81.8)	2616 (100)
Kiosks	31 (5.2)	563 (94.8)	594 (100)
Convenience stores	363 (29.0)	888 (71.0)	1251 (100)
*P-value**		*<0.001*	
Price/pack (GHC)			
Low price (2-7)	778 (61.2)	494 (38.8)	1272 (100)
High price (8-14)	93 (2.9)	3096 (97.1)	3189 (100)
P-value*		<0.001	
Cigarette brand (manufacturer)			
555 (BAT)	0 (0)	190 (100)	190(100)
London Brown/White (BAT)	0 (0)	928 (100)	928 (100)

For peer review only

Pallmall (BAT)	70 (14.2)	433 (85.8)	494 (100)
Business Royal (Independent Tobacco Inc)	210 (70.0)	90 (30.0)	300 (100)
Fine (unknown)	181 (78.3)	50 (21.6)	231 (100)
Rothmans Kingsize (BAT)	29 (1.6)	1798 (98.4)	1827 (100)
Oris (Oriental General Trading Inc)	107 (81.1)	35 (18.9)	132 (100)
Rothmans Royals (BAT)	99 (86.1)	20 (13.9)	115 (100)
Gold Seal (China Tobacco)	85 (91.4)	8 (8.6)	93 (100)
Tusker (BAT)	29 (100)	0 (0)	29 (100)
Others (Fisher, menthol, Cherry etc.)	61(50.0)	61 (50.0)	122 (100)
*P-value**	*<0.001*		

*P-value based on $\chi 2$ or Fisher's exact test

For the classification of illicit packs, majority were characterized by absence of tax stamps (94%), '*for sale in Ghana'* sign (92%), warning labels not in in English (77%) and absence of text and pictorial warning labels (28%).

Almost all the packs collected were the 20-stick pack (98%). The average price/pack of the 20-stick packs was 8.5 GHC and that for 10-stick was 3.3 GHC. Illicit packs had an average price/pack of 5.4 GHC (SD 1.5, range 2-12 GHC) whilst licit pack was 9.1 GHC (SD 2.1, range 2-14 GHC). Close to half of the illicit packs originated from Togo (51%), followed by Nigeria (15%) and then Cote d'Ivoire (10 %). About 2% of packs that were destined for Ghana were classified as illicit as the packs did not conform to the current labeling requirements as approved by FDA.

Table 2 shows the results from adjusted and unadjusted logistic regression of the factors associated with illicit cigarette sales in Ghana. The odds of illicit cigarette sales were 1.8 folds and 3.5 folds higher in convenience stores as compared to drinking bars in the unadjusted and adjusted models respectively (Table 2). Also, the sale of illicit cigarettes was 19.3 and 67.2 odds higher in border towns as compared to non-border towns in both the adjusted and unadjusted models respectively. The middle and coastal country zones had lower odds of illicit cigarettes sales than the northern zones in both the unadjusted and adjusted regression models respectively. Also, for every unit increase in price/pack, the odds of illicit cigarette consumption reduce by almost 60%.

For peer review only

Table 2: Unadjusted and adjusted factors for illicit cigarette sales in Ghana

Variable	Unadjusted		Adjusted	
	OR	95% CI	OR	95% CI
Retail shop type				
Drinking bars	1		1	
Kiosks	0.25	0.17 - 0.35	0.52	0.28-0.96
Convenience stores	1.83	1.57-2.15	3.47	1.92-6.26
Country Zone				
Northern	1		1	
Middle	0.03	0.01-0.05	0.42	0.16 -1.08
Coastal	0.54	0.46-0.63	0.70	0.39-1.25
Border/non border towns				
Non-border town	1		1	
Border town	19.3	16.0-23.4	67.2	(44.2-102.2)
Pack price	0.39<br>(coef= -0.94)	(0.37-0.42)<br>(-0.99 to -0.88)	0.39<br>(coef=-0.95)	(0.36 -0.42)<br>(-1.03 to -0.88)

Table 3 shows the results of bivariate and multivariate analysis adjusted for 384 vendors that collected packs from single stick sales. After adjusting for the clustering effect of vendors, convenience stores had higher odds of illicit cigarette sales in both the bivariate and multivariate analysis adjusted for vendors. Border towns also had higher odds of illicit in both bivariate and multivariate models

Table 3: Effect of clustering by vendors* on illicit cigarette sales

For peer review only

Variable	Bivariate		Multivariate	
	OR	95% CI	OR	95% CI
Retail shop type				
Drinking bars	1		1	
Kiosks	0.25	0.11 - 0.53	0.52	0.16-1.69
Convenience stores	1.83	1.03-3.26	3.47	1.22-9.84
Country Zone				
Northern	1		1	
Middle	0.03	0.01-0.08	0.42	0.01 -2.51
Coastal	0.54	0.30-0.95	0.70	0.22-2.27
Border/non border towns				
Non-border town	1		1	
Border town	19.3	8.80-42.40	67.2	(17.62-256.41)
Pack price	0.39 (coef= -0.94)	(0.31-0.50) (-0.99 to -0.89)	0.39 (coef=-0.95)	(0.32 -0.46) (-1.05 to -0.89)

*Adjusted for the clustering effect of vendors on illicit cigarette sales (n=384)

For peer review only

DISCUSSION

This study found out that close to 20% of the packs collected were illicit of the total 4461 packs. Majority of the illicit packs were reported from Aflao (Ghana-Togo border) (99%) and Tamale (46%). Tamale, although not a border town, is the capital of the Northern region of Ghana, and has most of the cigarettes smuggled from Burkina Faso (8). Close to half of the illicit packs originated from Togo (51%), followed by Nigeria (15%) and then Cote d'Ivoire (10%). The most common brand of cigarettes sold in Ghana was from BAT including Rothmans Kingsize, London Brown/White and Pall Mall. One out four of the illicit packs belonged to Business Royal (Independent Tobacco Company), a fifth were from Fine (unknown company) and about one out of ten were from Oris brand (Oriental General Trading). The most common features identified for classifying packs as illicit were the absence of tax stamps, '*for sale in Ghana'* sign and warning labels not in English. Adjusted and unadjusted logistic regression models indicated that convenience stores, border towns, northern zone of the country and price/pack had higher odds of illicit cigarettes consumption for single stick sales in Ghana.

Our study provides an objective measure and describes the nature of the illicit cigarette market. This plays a critical role in developing comprehensive and effective tobacco control policies, particularly in countries within sub-Saharan Africa such as Ghana, where data on illicit cigarettes sales is lacking. Our illicit cigarette estimates from single stick sales of 20%, is however, lower than the estimates of the Euromonitor (37% in 2018) (17), which is the only available estimate on illicit cigarettes market in Ghana. Nevertheless, the Euromonitor data is critisicied for lack of transparency and their and their funding source from the tobacco industry (TI) (17).

For peer review only

The TI is known for quoting high estimates of the illicit market as a means of deterring governments from imposing tobacco tax increases, which contributes to ineffective tobacco control and lost opportunities for the governments to collect more revenue.

There are various methods to assess the extent of illicit tobacco in any country, such as measuring the difference between consumption and tax paid sales (gap analysis), interviewing smokers, examination of littered cigarette packs and econometric modeling (18). We employed a methodology particularly suitable in countries with single stick sales, similar to methods used in India (9), Pakistan (19), Bangladesh (10) and Argentina (20). Despite a ban on single stick sales, all retailers (100%) sold single sticks, calling for enforcement of the ban. Our estimates of illicit cigarette sales (20%) are also similar to countries with a higher tobacco use prevalence such as Pakistan (18%) and Argentina (14%) that used a similar methodology (19,20). Despite the lack of estimates of illicit cigarettes from many countries in the African Region, countries such as South Africa, Kenya, The Gambia and Nigeria have available estimates of their illicit market using different methods of estimation. Our estimates were found to be lower than South Africa (with over 30% of the total market being illicit) (21), Nigeria (26%) (22) and Kenya (26%) (23) but higher than the Gambia (8.6%) (24). With the recent ratification of the Protocol in Ghana, and estimates suggesting one out of five cigarette packs to be illicit, there is an urgent need for governments to address this by fully implementing ratified protocol (which has specific requirements to improve traceability of tobacco products and increase tobacco industry accountability).

British American Tobacco (BAT) continues to dominate sales of cigarettes as evidenced by the most common cigarettes sold in Ghana (Rothmans Kingsize,

For peer review only

London Brown/White and Pall Mall). This is largely due to the company's long history in Ghana (25). While the company ceased domestic production in 2006, it remains the dominant importer of cigarettes into the country (25). There are also very low-priced brands available, such as BAT's Tusker brand (of which all packs were illicit). While, all packs from London Brown/White were found to be licit, about 14% of Pall Mall and 1.6% of Rothmans Kingsize were illicit, demonstrating the possibility of the industry's involvement in illicit trade (26). Further, the small-scale convenience stores were found to be a major selling point of illicit cigarettes. These are legally operating, widely available settings to the low-income Ghanaian smoker (who prefers to buy single stick) widely available in both rural and urban locations. Convenience stores were also found to have higher odds of illicit cigarette consumption as compared to drinking bars in both the adjusted and unadjusted logistic regression models, indicating that it may be an important predictor of illicit cigarette sales in the country.

Geography was found to play an important role in the illicit cigarette market in Ghana. A third of the packs collected from the northern zone of the country were found to be illicit. According to the Euromonitor (8), the north of Ghana sees particularly strong illicit trade, with most smuggling from Burkina Faso finding their way to this region into Tamale (17). This could also be linked to the high smoking prevalence and lower income population in the region as compared to other regions (27). Similarly, border towns were also found to be strong predictors of illicit cigarette sales. Six out of 10 packs collected from border towns were illicit and almost 100% of the packs collected from Aflao (Ghana-Togo border), and close to half of the packs from Tamale (large city in Northern Ghana linked to Burkina Faso) were found to be illicit. Border towns have been found to be more vulnerable to the trade of illicit

For peer review only

341 cigarette and tobacco products in Vietnam (28) and Georgia (29). Our findings reinforces the need for strengthening patrolling and border control in addition to building capacity and training for authorities belonging to customs, police and immigration. The illicit cigarettes originated from Togo (51%), followed by Nigeria (15%) and then Cote d'Ivoire (10%).

In terms of pricing of cigarettes, illicit packs were found to be almost 50% cheaper than licit packs. Africa in general, lags behind other regions (such as European and the Americas) in implementing strong tobacco tax policies (1). Close to 90% of the illicit packs were belonged to the low price category (2-7 GHC). Currently, the total excise tax on tobacco products in Ghana, accounts for only 31.8% of the average retail price (30). Also, over half of the smuggled cigarettes in the study originated from Togo where a pack of cigarettes is priced at about one USD and is about 0.50 USD in Ghana (30). The link between tobacco taxation and smuggling has been doubtful and inconsistent (31). According to a report by the World Bank (32), taxes and prices have only a limited impact on illicit cigarette market share at country level, contrary to arguments by the tobacco industry. The African region, with low prices and low taxation on tobacco products and high levels of smuggling, provides a good illustration of this observation. This calls for more research to understand the relationship between tobacco taxation and smuggling in Africa.

Our study findings should be considered in the light of some limitations. First, despite the wide geographical dispersion in the three zones of the country (northern, middle and coastal), the representativeness to the country is limited. Also, as data was collected during COVID-19 lockdown period in Ghana and we could not explore other border towns that were planned due to pertaining restrictions at that time. Secondly, the empty pack collection relies on retailers to provide us with all the

For peer review only

empty packs from previous day's single stick sales. It could be possible that some retailers would want to hide the illegal packs, which could underestimate our findings. Nevertheless, retailers were motivated with a monetary incentive, which, to an extent, mitigated this issue.

CONCLUSION

Our study found a total of 20% illicit packs in the entire sample of packs collected across the eight border and non-border towns/cities in Ghana. This study provides valuable information for policymakers and law enforcement in the region and bringing to light the inadequacy of the current monitoring and regulatory activities of the FDA and customs. Our findings have two important policy implications; first, the regulatory body and the focal point for tobacco control in Ghana (FDA) in collaboration with the customs, police and immigration, should strengthen the supply chain control and market surveillance at retail points in the towns and cities, particularly those close to the Ghana-Togo and Ghana-Burkina Faso border in the northern and coastal zones of the country, aside from border monitoring and transportation tracing. Secondly, with the introduction of Tax Stamp Policy since March 2018, Ghana should also consider the implementation of a supply chain control that resembles a track and trace system (like Kenya), independent of any industry influence to effectively monitor the illicit market.

ACKNOWLEDGEMENTS

We would also like to thank Michael Ababio and Christopher Bekoe for their role and contribution in pack collection and fieldwork. We would also like to thank all the retailers that provided information and packs for this survey.

CONTRIBUTORS

For peer review only

AS, FD, AG, TK, HR and EOD contributed to the design, conception, acquisition, analysis and interpretation of the project and data; the drafting and revision of the manuscript and the approval of the final version to be published. AS and DL contributed to the acquisition of data. LB contributed to the design and conception of the project. OB and AG contributed to the drafting and revision of the manuscript and the approval of the final version to be published.

COMPETING INTEREST

None declared

FUNDING

This work was supported by the Medical Research Council [grant number MR/P027946/2] with funding from the Global Challenges Research Fund and with additional funding from the University of Edinburgh's Scottish Funding Council Global Challenges Research Fund (GCRF) allocation.

Ethics Approval

The study protocol was approved by the Committee on Human Research, Publication and Ethics (Reference number: CHRPE/AP/441/18) and the University of Bath's Research Ethics Approval Committee for Health (REACH) (EP 19/20 063).

Data sharing statement

The data are owned and shared by the Tobacco Control Capacity Program (TCCP) and the School of Public Health, KNUST, Ghana. Requests for data sharing can be made to artisingh_uk@yahoo.com/arti.singh@tuni.fi

Legends for figures

Figure 1: Causal diagram of illicit cigarette consumption from single stick sales in Ghana (potential confounders were border towns, country zone, pack prices and type of retail shop)

For peer review only

Figure 2: Cigarette brands sold in Ghana

REFERENCES

1. WHO. Who Report on the Global Tobacco Epidemic, 2021. 2021;
2. Confronting illicit tobacco trade: a global review of country experiences.
3. Joossens L, Merriman D, Ross H et al IU against T and L. How eliminating the global illicit cigarette trade would increase tax revenue and save lives. 2009 [cited 2022 Feb 3];30. Available from: https://theunion.org/technical-publications/how-eliminating-the-global-illicit-cigarette-trade-would-increase-tax-revenue-and-save-lives
4. Transnational Alliance to Combat Illicit Trade. Mapping the Impact of Illicit Trade on the Sustainable Development Goals. 2019; Available from: www.tracit.org/publications.html
5. Singh A, Owusu-Dabo E, Mdege N, McNeill A, Britton J, Bauld L. A situational analysis of tobacco control in Ghana: progress, opportunities and challenges. Journal of Global Health Reports. 2020;
6. Tobacco in Ghana | Market Research Report | Euromonitor [Internet]. [cited 2021 Aug 17]. Available from: https://www.euromonitor.com/tobacco-in-ghana/report
7. Joossens L, Merriman D, Ross H, Raw M. The impact of eliminating the global illicit cigarette trade on health and revenue. Addiction. 2010 Sep 1;105(9):1640–9.
8. ICTD. Tobacco in Ghana | Market Research Report | Euromonitor [Internet]. [cited 2022 Jul 30]. Available from: https://www.euromonitor.com/tobacco-in-ghana/report
9. John RM, Ross H. Illicit cigarette sales in Indian cities: findings from a retail

For peer review only

survey. Tob Control. 2018 Nov 1;27(6):684–8.

10. Abdullah SM, Huque R, Bauld L, Ross H, Gilmore A, John RM, et al. Estimating the Magnitude of Illicit Cigarette Trade in Bangladesh: Protocol for a Mixed-Methods Study. International Journal of Environmental Research and Public Health 2020, Vol 17, Page 4791 [Internet]. 2020 Jul 3 [cited 2022 Jan 5];17(13):4791. Available from: https://www.mdpi.com/1660-4601/17/13/4791/htm

11. Michal S, Guillermo P, Blecher E. A Toolkit on Measuring Illicit Trade in Tobacco Products. 2020;

12. Welcome to Ghana Food And Drugs Authority | Home [Internet]. [cited 2022 Jan 5]. Available from: http://www.fdaghana.gov.gh/index.php

13. Campaign for Tobacco-Free Kids. Ghana Details | Tobacco Control Laws [Internet]. 2022 [cited 2022 Jul 30]. Available from: https://www.tobaccocontrollaws.org/legislation/country/ghana/pl-health-warnings

14. GRA introduces Ghana Tax Stamp Authenticator mobile app [Internet]. [cited 2022 Feb 23]. Available from: https://www.ghanaweb.com/GhanaHomePage/business/GRA-introduces-Ghana-Tax-Stamp-Authenticator-mobile-app-771674

15. Vellios N, Van Walbeek C, Ross H. Measuring the illicit cigarette market in the absence of pack security features: A case study of South Africa. Tobacco Control. 2021 Feb 25;

16. John RM, Ross H. Consumption of illicit cigarettes in India: findings from a retail survey. Tobacco Induced Diseases. 2018;

17. Profile I. Tobacco in. 2011;(October):1–32.

For peer review only

18. A Toolkit on Measuring Illicit Trade in Tobacco Products | Tobacconomics [Internet]. [cited 2022 Jan 5]. Available from: https://tobacconomics.org/research/a-toolkit-on-measuring-illicit-trade-in-tobacco-products/

19. Khan A, Dobbie F, Siddiqi K, Ansaari S, Abdullah SM, Iqbal R, et al. Illicit cigarette trade in the cities of Pakistan: comparing findings between the consumer and waste recycle store surveys. Tobacco Control [Internet]. 2021 Apr 15 [cited 2021 Dec 30];1–7. Available from: https://tobaccocontrol.bmj.com/content/early/2021/04/14/tobaccocontrol-2020-056386

20. Pizarro ME, Giacobone G, Shammah C, Stoklosa M. Illicit tobacco trade: empty pack survey in eight Argentinean cities. Tobacco Control [Internet]. 2021 Apr 8 [cited 2021 Dec 30];0:1–7. Available from: https://tobaccocontrol.bmj.com/content/early/2021/04/07/tobaccocontrol-2020-056405

21. Vellios N, van Walbeek C, Ross H. Illicit cigarette trade in South Africa: 2002–2017. Tobacco Control [Internet]. 2020 Oct 1 [cited 2021 Dec 30];29(Suppl 4):s234–42. Available from: https://tobaccocontrol.bmj.com/content/29/Suppl_4/s234

22. Adeniran AP, Ekeruche MA, Castradori M. Illicit Tobacco in Nigeria [Internet]. Centre for the Study of the Economies of Africa (CSEA); 2020 [cited 2021 Dec 23]. Available from: https://www.africaportal.org/publications/illicit-tobacco-nigeria/

23. Ross H. Tracking and tracing tobacco products in Kenya. Preventive Medicine. 2017 Dec 1;105:S15–8.

For peer review only

24. Chisha Z, Janneh ML, Ross H. Consumption of legal and illegal cigarettes in the Gambia. Tob Control [Internet]. 2020;29:254–9. Available from: http://tobaccocontrol.bmj.com/

25. Owusu-Dabo E, Lewis S, McNeill A, Anderson S, Gilmore A, Britton J. Smoking in Ghana: A review of tobacco industry activity. Tobacco Control. 2009;

26. Morris P. British American Tobacco Fights Dirty In West Africa British American Tobacco Fights Dirty In West Africa. 2021;

27. Yawson AE, Baddoo A, Hagan-Seneadza NA, Calys-Tagoe B, Hewlett S, Dako-Gyeke P, et al. Tobacco use in older adults in Ghana: Sociodemographic characteristics, health risks and subjective wellbeing. BMC Public Health. 2013;

28. Nguyen HTT, Giang LT, Pham TN. Empirical analysis on the illicit trade of cigarettes in Vietnam. Tobacco Control [Internet]. 2020 Oct 1 [cited 2021 Dec 30];29(Suppl 4):s281–6. Available from: https://tobaccocontrol.bmj.com/content/29/Suppl_4/s281

29. Little M, Ross H, Bakhturidze G, Kachkachishvili I. Illicit tobacco trade in Georgia: prevalence and perceptions. Tobacco Control [Internet]. 2020 Oct 1 [cited 2021 Dec 30];29(Suppl 4):s227–33. Available from: https://tobaccocontrol.bmj.com/content/29/Suppl_4/s227

30. HealthBridge. Tobacco Taxation Factsheet [Internet]. 2020. Available from: https://healthbridge.ca/images/uploads/library/Factsheet_2_-_Taxation_final.pdf

31. Economic WA. Tobacco Taxation Factsheet : Smoking in Mali : The Facts Tobacco Taxation in Mali. 2019;2019–20.

For peer review only

32. World Bank. Confronting Llicit Tobacco Trade. 2019; Available from: https://thedocs.worldbank.org/en/doc/662791548435048907-0090022019/original/WBGTobaccoIllicitTradeSACU.pdf

For peer review only

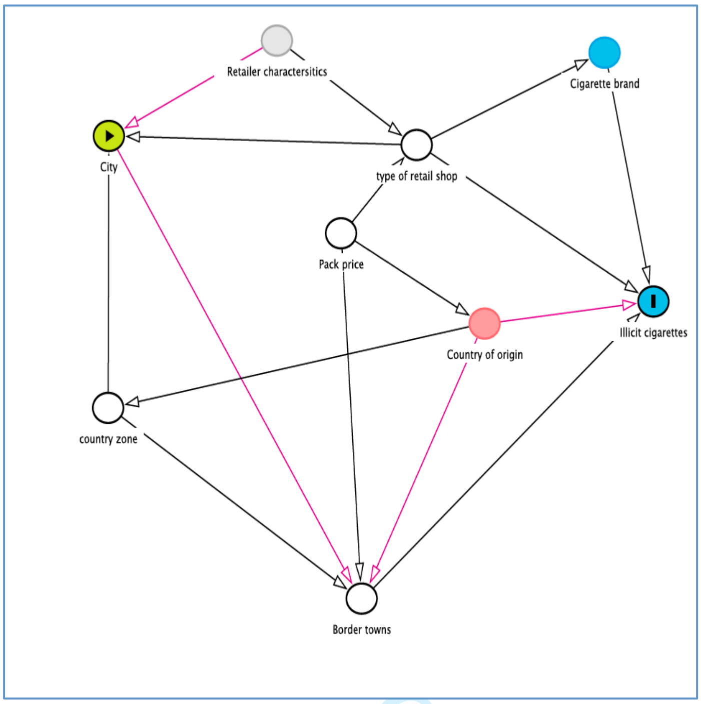

Figure 1: Causal diagram of illicit cigarette consumption from single stick sales in Ghana (potential confounders were border towns, country zone, pack prices and type of retail shop)

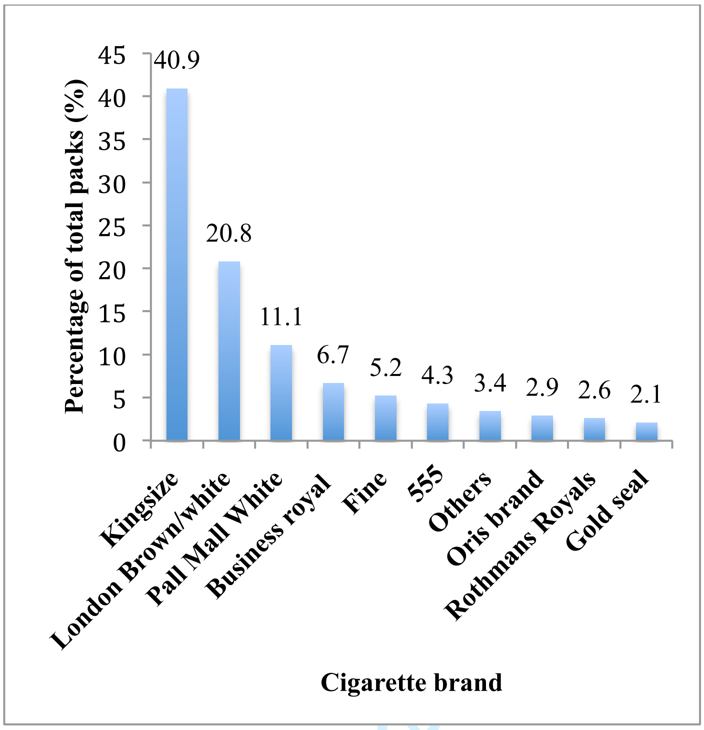

Figure 2: Common cigarette brands sold in Ghana

STROBE Statement—Checklist of items that should be included in reports of ***cross-sectional studies***

	Item No	Recommendation	Page No
**Title and abstract**	1	(*a*) Indicate the study's design with a commonly used term in the title or the abstract	Title and abstract
		(*b*) Provide in the abstract an informative and balanced summary of what was done and what was found	Page 2
**Introduction**			
Background/rationale	2	Explain the scientific background and rationale for the investigation being reported	Page 4-5
Objectives	3	State specific objectives, including any prespecified hypotheses	Page 5
**Methods**			
Study design	4	Present key elements of study design early in the paper	Page 5-6
Setting	5	Describe the setting, locations, and relevant dates, including periods of recruitment, exposure, follow-up, and data collection	Page 5-6
Participants	6	(*a*) Give the eligibility criteria, and the sources and methods of selection of participants	Page 6-7
Variables	7	Clearly define all outcomes, exposures, predictors, potential confounders, and effect modifiers. Give diagnostic criteria, if applicable	Page 7-8
Data sources/ measurement	8*	For each variable of interest, give sources of data and details of methods of assessment (measurement). Describe comparability of assessment methods if there is more than one group	Page 7-8
Bias	9	Describe any efforts to address potential sources of bias	Page 7-8
Study size	10	Explain how the study size was arrived at	Page 6
Quantitative variables	11	Explain how quantitative variables were handled in the analyses. If applicable, describe which groupings were chosen and why	Page 7-8
Statistical methods	12	(*a*) Describe all statistical methods, including those used to control for confounding	Page 7-8
		(*b*) Describe any methods used to examine subgroups and interactions	N/A
		(*c*) Explain how missing data were addressed	Page 7
		(*d*) If applicable, describe analytical methods taking account of sampling strategy	N/A
		(*e*) Describe any sensitivity analyses	N/A
**Results**			
Participants	13*	(a) Report numbers of individuals at each stage of study—eg numbers potentially eligible, examined for eligibility, confirmed eligible, included in the study, completing follow-up, and analysed	Pages 8-10
		(b) Give reasons for non-participation at each stage	N/A
		(c) Consider use of a flow diagram	N/A
Descriptive data	14*	(a) Give characteristics of study participants (eg demographic, clinical, social) and information on exposures and potential confounders	Pages 8-10
		(b) Indicate number of participants with missing data for each variable of interest	N/A
Outcome data	15*	Report numbers of outcome events or summary measures	Pages 10-11
Main results	16	(*a*) Give unadjusted estimates and, if applicable, confounder-adjusted estimates and their precision (eg, 95% confidence interval). Make clear which confounders were adjusted for and why they were included	Pages10-11

For peer review only - http://bmjopen.bmj.com/site/about/guidelines.xhtml

		(*b*) Report category boundaries when continuous variables were categorized	Pages 10-11
		(*c*) If relevant, consider translating estimates of relative risk into absolute risk for a meaningful time period	N/A
Other analyses	17	Report other analyses done—eg analyses of subgroups and interactions, and sensitivity analyses	N/A
**Discussion**			
Key results	18	Summarise key results with reference to study objectives	Page 11-12
Limitations	19	Discuss limitations of the study, taking into account sources of potential bias or imprecision. Discuss both direction and magnitude of any potential bias	Page 15-16
Interpretation	20	Give a cautious overall interpretation of results considering objectives, limitations, multiplicity of analyses, results from similar studies, and other relevant evidence	Page 12-15
Generalisability	21	Discuss the generalisability (external validity) of the study results	Page 15
**Other information**			
Funding	22	Give the source of funding and the role of the funders for the present study and, if applicable, for the original study on which the present article is based	Page 17

*Give information separately for exposed and unexposed groups.

Note: An Explanation and Elaboration article discusses each checklist item and gives methodological background and published examples of transparent reporting. The STROBE checklist is best used in conjunction with this article (freely available on the Web sites of PLoS Medicine at http://www.plosmedicine.org/, Annals of Internal Medicine at http://www.annals.org/, and Epidemiology at http://www.epidem.com/). Information on the STROBE Initiative is available at www.strobe-statement.org.

For peer review only

For peer review only - http://bmjopen.bmj.com/site/about/guidelines.xhtml
